# Genome-wide association mapping for root traits in a panel of rice accessions from Vietnam

**DOI:** 10.1186/s12870-016-0747-y

**Published:** 2016-03-10

**Authors:** Nhung Thi Phuong Phung, Chung Duc Mai, Giang Thi Hoang, Hue Thi Minh Truong, Jeremy Lavarenne, Mathieu Gonin, Khanh Le Nguyen, Thuy Thi Ha, Vinh Nang Do, Pascal Gantet, Brigitte Courtois

**Affiliations:** Agricultural Genetics Institute, National Key Laboratory for Plant Cell Biotechnology, LMI RICE, 00000 Hanoi, Vietnam; University of Science and Technology of Hanoi, LMI RICE, 00000 Hanoi, Vietnam; IRD, LMI RICE, 00000 Hanoi, Vietnam; Université de Montpellier, UMR DIADE, 34095 Montpellier, France; Cirad, UMR-AGAP, 34398 Montpellier, France

**Keywords:** Rice, Genotyping by sequencing, Root development, Association mapping, Structure

## Abstract

**Background:**

Despite recent sequencing efforts, local genetic resources remain underexploited, even though they carry alleles that can bring agronomic benefits. Taking advantage of the recent genotyping with 22,000 single-nucleotide polymorphism markers of a core collection of 180 Vietnamese rice varieties originating from provinces from North to South Vietnam and from different agrosystems characterized by contrasted water regimes, we have performed a genome-wide association study for different root parameters. Roots contribute to water stress avoidance and are a still underexploited target for breeding purpose due to the difficulty to observe them.

**Results:**

The panel of 180 rice varieties was phenotyped under greenhouse conditions for several root traits in an experimental design with 3 replicates. The phenotyping system consisted of long plastic bags that were filled with sand and supplemented with fertilizer. Root length, root mass in different layers, root thickness, and the number of crown roots, as well as several derived root parameters and shoot traits, were recorded. The results were submitted to association mapping using a mixed model involving structure and kinship to enable the identification of significant associations. The analyses were conducted successively on the whole panel and on its indica (115 accessions) and japonica (64 accessions) subcomponents. The two associations with the highest significance were for root thickness on chromosome 2 and for crown root number on chromosome 11. No common associations were detected between the indica and japonica subpanels, probably because of the polymorphism repartition between the subspecies. Based on orthology with *Arabidopsis*, the possible candidate genes underlying the quantitative trait loci are reviewed.

**Conclusions:**

Some of the major quantitative trait loci we detected through this genome-wide association study contain promising candidate genes encoding regulatory elements of known key regulators of root formation and development.

**Electronic supplementary material:**

The online version of this article (doi:10.1186/s12870-016-0747-y) contains supplementary material, which is available to authorized users.

## Background

Vietnam is a tropical country in Southeast Asia with a rice-based agricultural economy. Rice is grown on 82 % of the agricultural area, which corresponds to 7.75 M ha for a production of 43.6 million tons in 2012 [1]. Vietnam is the world’s second rice exporter (6.4 million tons in 2012). Rice is mainly grown under irrigated conditions in the river deltas, notably the Mekong delta in South Vietnam (52 % of Vietnam rice production) and the Red River delta in North Vietnam (18 % of Vietnam rice production); however, because three-quarters of Vietnam’s territory is made up of mountainous and hilly regions, other ecosystems are also represented (upland, rainfed lowland and mangrove).

Vietnam is among countries most threatened by climate change [2]. In particular, between spring and summer, all of the central areas of Vietnam are subject to periods of recurrent and severe drought that affect rice plantlets just after planting or plants during grain filling and can result in important yield losses. To improve rice drought resistance, an ideotype with a large number of deep and thick roots and a high root-to-shoot ratio was advocated, assuming that there was water at depth in the soil profile [3]. However, because roots develop underground and are not easily observed, this ideotype is difficult to select for. One way to achieve this goal would be to use indirect selection based on markers that are tightly linked to genes that control these root traits [4]. Knowledge of the genetic control of root development in rice is rapidly improving. Numerous root quantitative trait loci (QTLs) have been detected in various mapping populations ([5] for a review). Three QTLs that are involved in water and nutrient uptake by roots have recently been cloned [6–8]. Furthermore, other QTLs have been finely mapped, and the underlying genes are close to being identified [9, 10]. The rice orthologs of several genes that were initially identified in *Arabidopsis* have also been shown to have an effect on root development in rice (reviewed in [11–14]). However, this useful information is still far from giving a clear overall pattern of the network of genes that are involved. Genome-wide association studies (GWAS) are a way to directly identify new candidate genes or, more reasonably, to narrow down the chromosomal segments that carry functional factors to much smaller intervals [15]. Because of the lower linkage disequilibrium (LD) that is encountered in natural populations, the resolution of QTL detection in such populations is higher than that obtained by classical mapping populations of the same size. However, the corollary of this low LD is that the average distance between the markers that are used to genotype the population needs to be shorter than the LD decay distance to properly cover the whole genome. Such high marker density has only become accessible, in most species, with the development of new sequencing technologies, notably genotyping by sequencing (GBS). Genotyped panels representing a broad geographic diversity have been developed [16, 17] and used in GWAS for root traits [17, 18]. However, although their size is on the order of 150 to 400 accessions, these panels still explore only a small fraction of the large rice diversity. Accessions from Vietnam are not widely represented in world-wide panels although local genetic resources, notably from geographically diverse countries, have been shown to bear unexploited but interesting variations for useful traits [19, 20]. Even among the 3000 rice genomes that were recently sequenced, only 55 Vietnamese accessions were included [21]. To take advantage of the allelic richness that can be encountered locally, we have developed a panel that is exclusively composed of accessions from Vietnam (Additional file 1: Table S1). This panel of 182 accessions has been genotyped with approximately 22,000 single nucleotide polymorphisms (SNPs) using GBS, and its structure and the decay of LD have been analyzed in depth [22]. The panel is composed of two-thirds indica, one-third japonica and a few admixed accessions. Several subpopulations (6 in the indica subpanel and 4 in the japonica one) were detected within each subpanel. The average distances between polymorphic markers are 18 kb, 28 kb and 44 kb, for the whole panel, the indica and the japonica subpanels, respectively. On average, the pairwise LD, measured by r2, reaches 0.52 and 0.71 at 25 kb in the indica and japonica subpanels, respectively, and decays faster to background levels in the indica subpanel (*r*2 < 0.2 at 100 kb) than in the japonica subpanel (*r*2 < 0.2 at 425 kb). Because the distance between markers is shorter than the LD decay, the marker coverage is sufficient to undertake GWAS in all panels. Because the accessions came from different ecosystems, ranging from upland to mangrove, that were subject to specific but severe stresses (e.g., drought for upland or rainfed lowland rice or salinity for irrigated or mangrove rice), this panel constitutes an excellent resource for studying the genetic control of root system architecture and abiotic stress resistance.

In this paper, we performed an association study on root traits using our panel of Vietnamese varieties. Using a soil column system, different root parameters (maximum root depth, root biomass in different soil layers, crown root number, and crown root thickness) were investigated. Several QTLs were detected in the indica and japonica subpanels or in the whole panel. Among these QTLs, one associated with crown root thickness on chromosome 2 and one associated with crown root number on chromosome 11 had the highest levels of significance.

## Results

### Phenotyping

**Fig. 1:**
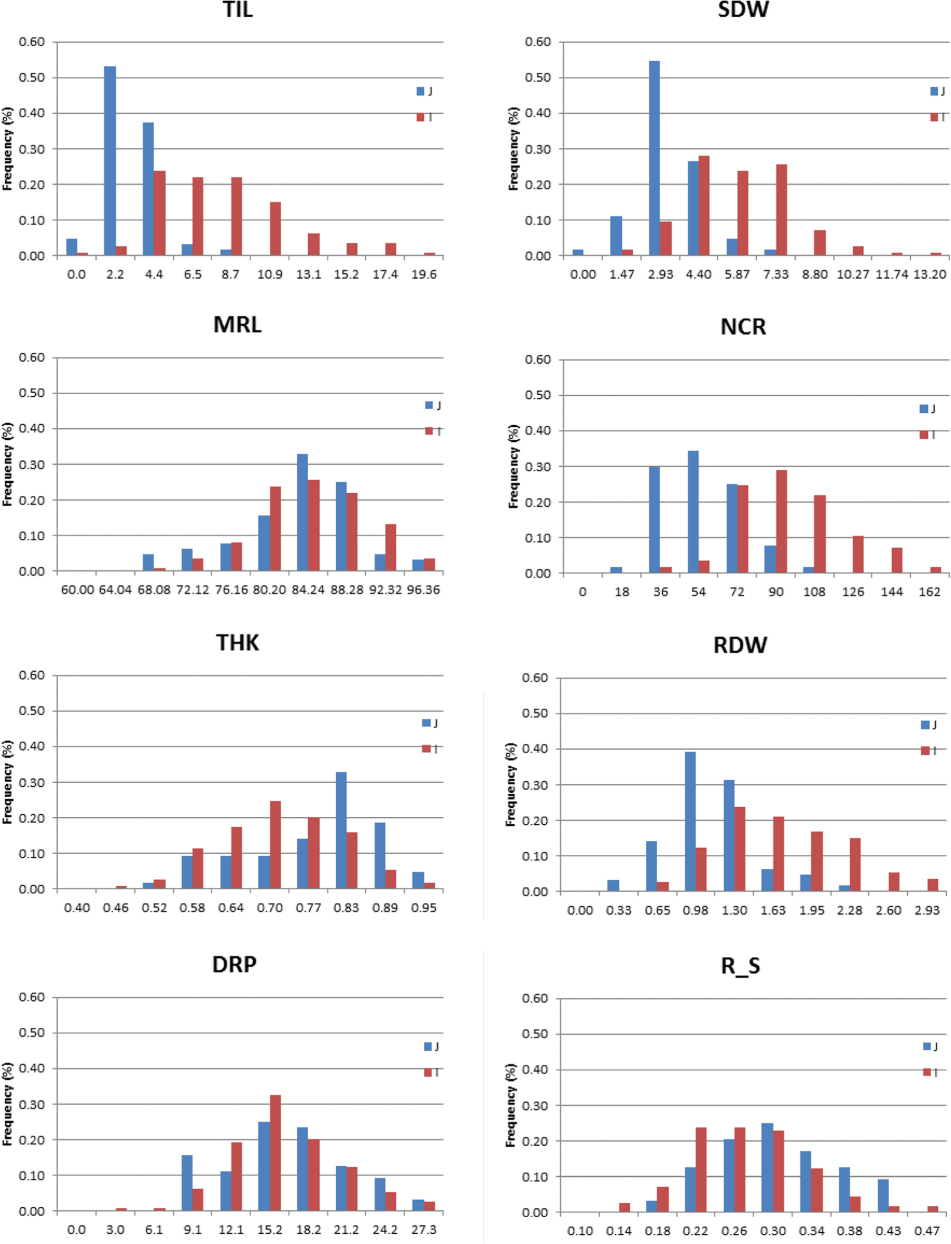
Frequency of distribution per subpanel for selected traits. In blue japonica subpanel; in red indica subpanel. TIL = number of tillers; SDW = shoot dry weight; MRL = maximum root length; NCR = number of crown roots; THK = root thickness; RDW = root dry weight; DRP = deep root proportion; R_S = root to shoot ratio

The results of the analysis of variance (ANOVA) are presented in Table 1. The variety effect was highly significant for all of the traits. The broad-sense heritability of the traits, ranging from 0.65 to 0.90, was moderate to high, with the exception of two related traits (deepest point reached by roots (DEPTH) and maximum root length (MRL)) for which values of 0.35 and 0.46, respectively, were registered. The replication effect was often significant, and the block effect was almost always highly significant, indicating some internal heterogeneity within replicates that the design helped to control. This environmental heterogeneity may be due to slight differences in light intensity due to the shade from neighbor trees and to the disposition of the blocks in the screenhouse, some peripheral, some central. The accession means were therefore adjusted from block effects. The mean, standard deviation, range and coefficient of variation (CV) of the whole panel are presented in Additional file 2: Table S2. A graphical representation of the plant architecture of each accession is shown in Additional file 3: Figure S1. A moderate to large variation was observed for most of the traits, as seen through the CVs of the panel varying from 20 % to 63 %, with the exception of longest leaf length (LLGHT), DEPTH, MRL, root thickness (THK) and shallow root proportion (SRP) whose CVs were less than 20 %. The same elements for the indica and japonica subpanels are presented in Table 2. For most of the shoot and root biomass traits, including deep root traits (shoot dry weight (SDW), MRL, root mass in the 00–20 cm segment (DW0020), root mass in the 20–40 cm segment (DW2040), root mass in the 40–60 cm segment (DW4060), root mass below 60 cm (DWB60), root dry weight (RDW), deep root mass (<40 cm) weight (DRW) and plant dry weight (PDW)), the mean values of the indica accessions were higher than those of the japonica accessions. The indica accessions had on average a much larger biomass, shorter leaves, more tillers and many more crown roots but had thinner roots and fewer resources allocated to roots, notably to deep roots (lower root to shoot ratio (R_S) and slightly lower deep root proportion (<40 cm) (DRP)). However, the trait distributions (Fig. 1) showed that the range of variation of the indica and japonica accessions was largely overlapping. To confirm these results and assess to what extent the observed phenotypic variability was determined by the genetic structure, a mean comparison was conducted between groups within the whole panel and between subpopulations within each subpanel for the genotyped accessions (Additional file 4: Table S3). For the majority of the traits except for DEPTH, MRL, DWB60, SRP, DRP and R_S, the phenotypic differences between the indica and japonica subpanels within the whole panel were highly significant. There were also difference between subpopulations within each subpanel for most of the traits except for DEPTH for the indica subpanel, number of tillers (TIL), SDW, number of crown root per tiller (NR_T) and PDW for the japonica subpanel and DW0020 for both subpanels. The percentage of phenotypic variance that was explained by the panel structure, which provides an alternate estimate of the relationships between genetic structure and phenotype for a given trait, gave similar results, with high percentages generally associated with the highest within-subpanel phenotypic differentiation (Additional file 4: Table S3). The mean comparisons showed that subpopulations I3 and, to a lesser extent, I6 in the indica subpanel and subpopulations J1 and J3 in the japonica subpanel had the deepest and thickest roots while subpopulations I1 and I5 as well as J2 and J4 registered the poorest performances in this respect.

**Table 1:** Result of the analysis of variance and trait broad sense heritability

Trait	Rep	Block(Rep)	Variety	h2
LLGHT	<0.001	< 0.001	< 0.001	0.90
TIL	< 0.001	0.0009	< 0.001	0.80
SDW	0.0043	< 0.0001	< 0.0001	0.73
DEPTH	0.0254	0.0003	0.0002	0.35
MRL	0.1428	0.0277	0.0001	0.46
NCR	0.2270	< 0.0001	< 0.0001	0.84
NR_T	<0.001	0.3450	< 0.0001	0.72
THK	0.0071	0.0017	< 0.0001	0.84
DW0020	0.0546	< 0.0001	< 0.0001	0.74
DW2040	0.1605	< 0.0001	< 0.0001	0.68
DW4060	0.4307	< 0.001	< 0.0001	0.69
DWB60	0.0260	0.0047	< 0.0001	0.70
DRW	0.0863	0.0004	< 0.0001	0.75
RDW	0.0650	< 0.0001	< 0.0001	0.75
PDW	0.0364	< 0.0001	< 0.0001	0.73
SRP	0.0207	0.0002	< 0.0001	0.72
DRP	0.0179	0.0045	< 0.0001	0.65
R_S	< 0.0001	< 0.0001	< 0.0001	0.75

*Rep* replication, *LLGTH* longest leaf length, *TIL* number of tillers, *SDW* shoot dry weight, *DEPTH* deepest point reached by roots, *MRL* maximum root length, *NCR* number of crown roots, *NR_T* number of crown root per tiller, *THK* root thickness, *DW0020* root mass in the 00–20 cm segment, *DW2040* root mass in the 20–40 cm segment, *DW4060* root mass in the 40–60 cm segment, *DWB60* root mass below 60 cm, *DRW* deep root mass (<40 cm) weight, *RDW* root dry weight, *PDW* plant dry weight, *SRP* shallow root proportion (0–20 cm), *DRP* deep root proportion (<40 cm), *R_S* root to shoot ratio

**Table 2:** Adjusted mean, standard deviation (sd), range, and coefficient of variation (CV) of the indica (ind) and japonica (jap) sub-panels for all traits

Traits	N	N	Mean	sd	Min	Max	CV	Mean	sd	Min	Max	CV
	ind	jap	ind	ind	ind	ind	ind	jap	jap	jap	jap	jap
LLGHT (cm)	121	66	93.3	12.8	63.9	116.4	13.7	97.7	11.9	67.3	125.0	12.2
TIL	121	66	9.18	3.62	1.61	20.75	39.5	4.27	1.51	1.69	10.83	35.6
SDW (g)	121	66	6.540	2.058	2.230	13.67	31.5	4.074	1.138	1.283	7.785	27.9
DEPTH (cm)	121	66	69.0	3.9	58.8	76.8	5.6	69.4	4.4	53.4	76.10	6.4
MRL (cm)	121	66	86.2	5.8	69.6	99.4	6.7	85.3	6.2	70.3	97.18	7.2
NCR	121	66	105.6	25.1	42.8	176.8	23.7	64.9	18.4	32.5	119.1	28.3
NR_T	121	66	13.6	4.2	5.5	26.9	30.9	16.1	4.3	7.8	34.9	26.8
THK (mm)	121	66	0.747	0.096	0.488	0.986	12.9	0.815	0.107	0.568	0.999	13.1
DW0020 (g)	121	66	0.991	0.267	0.410	1.785	26.9	0.682	0.177	0.313	1.290	26.0
DW2040 (g)	121	66	0.500	0.177	0.129	1.025	35.4	0.371	0.122	0.128	0.686	32.9
DW4060 (g)	121	66	0.237	0.104	0.034	0.549	43.9	0.156	0.069	0.035	0.349	44.2
DWB60 (g)	121	66	0.102	0.065	0.009	0.364	64.1	0.087	0.051	−0.005	0.197	58.2
DRW (g)	121	66	0.339	0.154	0.059	0.780	45.3	0.243	0.108	0.031	0.464	44.6
RDW (g)	121	66	1.830	0.549	0.714	3.164	30.0	1.295	0.369	0.472	2.284	28.4
PDW (g)	121	66	8.360	2.493	3.033	16.81	29.8	5.370	1.440	1.936	10.06	26.8
SRP (%)	121	66	55.5	6.6	42.7	83.1	11.9	53.4	6.5	37.7	67.4	12.2
DRP (%)	121	66	17.8	4.4	4.5	29.3	24.6	18.2	4.9	9.1	28.4	26.7
R_S	121	66	0.294	0.061	0.170	0.497	20.7	0.329	0.061	0.212	0.459	18.5

*LLGTH* longest leaf length, *TIL* number of tillers, *SDW* shoot dry weight, *DEPTH* deepest point reached by roots, *MRL* maximum root length, *NCR* number of crown roots, *NR_T* number of crown root per tiller, *THK* root thickness, *DW0020* root mass in the 00–20 cm segment, *DW2040* root mass in the 20–40 cm segment, *DW4060* root mass in the 40–60 cm segment, *DWB60* root mass below 60 cm, *DRW* deep root mass (<40 cm) weight, *RDW* root dry weight, *PDW* plant dry weight, *SRP* shallow root proportion (0–20 cm), *DRP* deep root proportion (<40 cm), *R_S* root to shoot ratio

The correlation coefficients among traits were highly significant and similar in direction within the whole panel and the two subpanels (Additional file 5: Table S4). The magnitude of the differences between the indica and japonica subpanels varied from trait to trait but was generally small, except for combinations involving number of crown roots (NRC). The high positive correlations between root dry masses in different layers (greater than 0.8; data not shown) were derived from their pyramidal relationships. NCR was highly correlated with TIL (0.72 in the whole panel), as expected because the root and tiller emissions are synchronized in rice. To determine whether it was possible to disentangle these two traits, the NR/T ratio was calculated. TIL, NCR and NR/T were not correlated with the root depth (whether MRL or LENGTH).

**Fig. 2:**
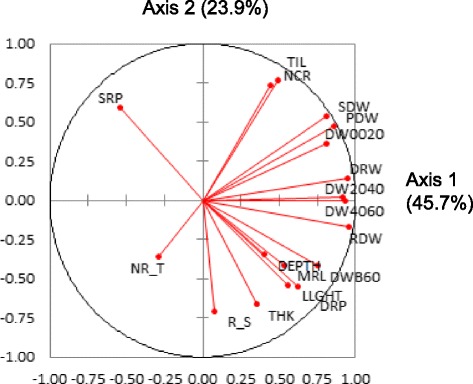
Circle of correlations for a PCA conducted on the whole panel and 18 traits. LLGTH = longest leaf length; TIL = number of tillers; SDW = shoot dry weight; DEPTH = deepest point reached by roots; MRL = maximum root length; NCR = number of crown roots; NR_T = number of crown root per tiller; THK = root thickness; DW0020 = root mass in the 00-20 cm segment; DW2040 = root mass in the 20-40 cm segment; DW4060 = root mass in the 40-60 cm segment; DWB60 = root mass below 60 cm; DRW = deep root mass (<40 cm) weight; RDW = root dry weight; PDW = plant dry weight, SRP = shallow root proportion (0–20 cm); DRP = deep root proportion (<40 cm); R_S = root to shoot ratio

**Fig. 3:**
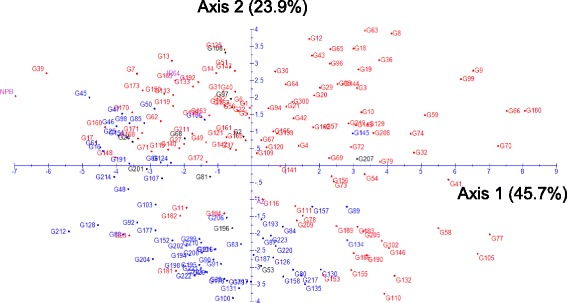
Scatterplot of the accessions of the whole panel based on a PCA on the phenotypic data (18 traits). Indica in red; japonica in blue; check in pink; intermediates in black. Axis 1 and axis 2 explains 45.7 % and 23.9 % of the variation respectively

A principal components analysis (PCA) was run on the adjusted means of all of the accessions. Together, the two first axes of the PCA explained 69.6 % of the variation. As shown by the circle of correlations (Fig. 2), almost all traits, with the exception of SRP and NR_T, which are ratios, were positively correlated with axis 1. Axis 1 can be viewed as an axis of increasing vigor opposing small and large plants when examining the accession positions on the first plane (Fig. 3). R_S was the only trait not correlated to axis 1. The second axis was characterized by an opposition between TIL, NCR, PDW, SDW, SRP and DW0020, corresponding to superficial biomass, and DEPTH, MRL, DRP and DWB60, corresponding to root biomass in the deepest layer. Root biomass in the intermediate layers (DW2040 and DW4060) was not correlated to axis 2. R_S, THK and LLGTH were also strongly correlated to axis 2, indicating that deep rooted varieties had also thick roots, long leaves and a high root to shoot ratio, all features that are characteristics of the tropical japonica group. The distribution of the accessions on the first plane (Fig. 3) confirmed these interpretations. The two top and the bottom-right quadrants were mostly occupied by indica accessions (in red), while japonica accessions (in blue) were mostly found in the lower-left quadrant, showing a much clearer separation than when considering each trait separately. However, the indica and japonica clouds overlapped to some extent, and some indica accessions were found in the middle of the japonica accessions and vice versa. When repeated for the indica and japonica panels separately, the patterns were highly similar to that of the whole panel (data not shown).

### Association mapping

**Fig. 4:**
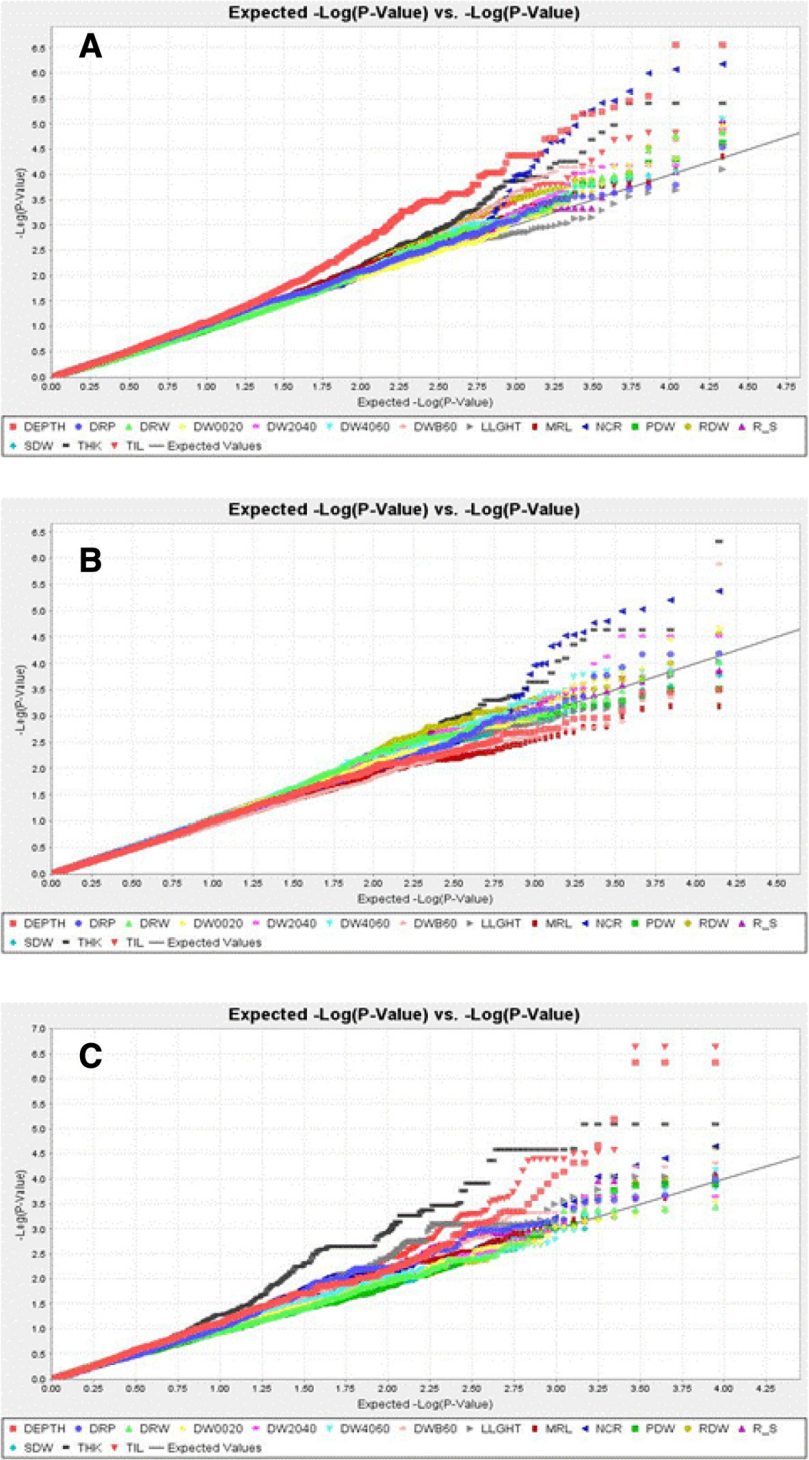
Quantile-quantile plots for the whole panel (**a**), the indica (**b**) and the japonica (**c**) subpopulations. The different traits are represented by different colors. The black diagonal represents the uniform law. LLGTH = longest leaf length; TIL = number of tillers; SDW = shoot dry weight; DEPTH = deepest point reached by roots; MRL = maximum root length; NCR = number of crown roots; NR_T = number of crown root per tiller; THK = root thickness; DW0020 = root mass in the 00–20 cm segment; DW2040 = root mass in the 20–40 cm segment; DW4060 = root mass in the 40–60 cm segment; DWB60 = root mass below 60 cm; DRW = deep root mass (<40 cm) weight; RDW = root dry weight; PDW = plant dry weight, SRP = shallow root proportion (0–20 cm); DRP = deep root proportion (<40 cm); R_S = root to shoot ratio

We performed successive association mappings for the whole panel and then separately for the indica and japonica subpanels. The mixed model that included both the structure and kinship matrices exerted good control over false positive rates for most traits as shown by the quantile-quantile plots for the whole set of accessions, the indica set and the japonica set, respectively (Fig. 4a to c). On these graphs, for most traits, the cumulative distribution of observed *P*-values fitted well with the expected uniform distribution that was represented by the diagonal, at least for the smallest log (*P*-values). There were two exceptions, DEPTH for the whole panel and THK for the japonica subpanel, for which the curves moved away from the diagonal. The inflation factor lambda was computed to quantitatively assess the extent of these deviations. Lambda was in the range of 0.95 to 1.07 for all traits except these two (1.25 for DEPTH in the whole panel and 1.50 for THK in the japonica subpanel, respectively). For these two trait x panel combinations, a larger number of false positives is expected compared with other combinations.

**Fig. 5:**
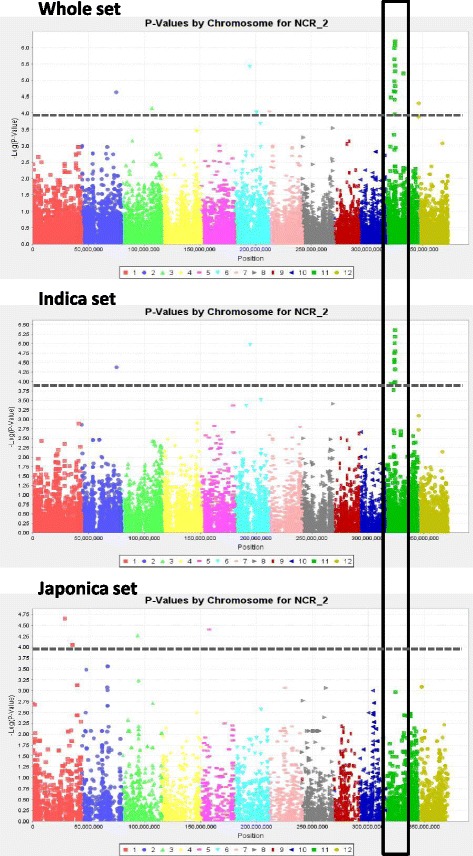
Manhattan plots for number of crown roots for the whole panel and the two sub-panels (the horizontal dotted line corresponds to *P* = 1e-04)

**Fig. 6:**
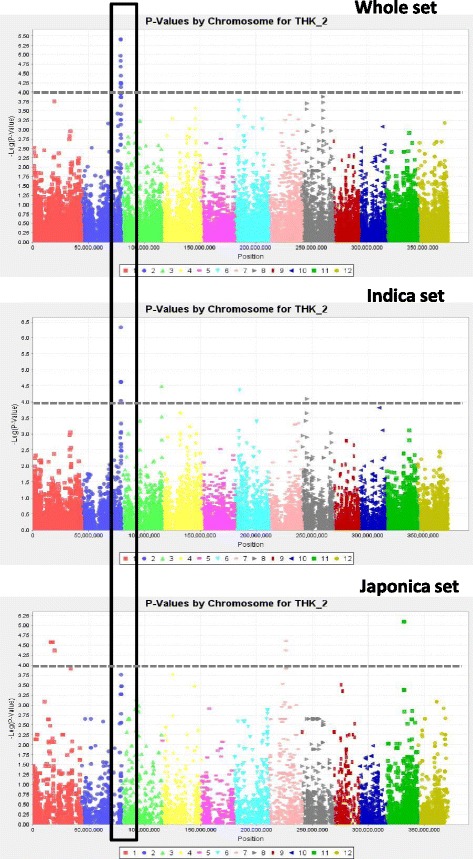
Manhattan plots for root thickness for the whole panel and the two sub-panels

In the whole panel, and the indica and japonica subpanels, 66, 20 and 26 markers, respectively, were significant at P ≤ 1e-04 (Table 3). The higher number of QTLs that were detected in the whole panel is most likely the result of its larger size. The most significant associations were recorded for DEPTH on chromosome 1 (q17; *P* = 2.67e-07) and NCR on chromosome 11 (q45; *P* = 6.59e-07) for the whole panel, THK on chromosome 2 (q57; *P* = 4.77e-07) for the indica subpanel, and TIL on chromosome 1 (q4; *P* = 2.28e-07) and DEPTH on chromosome 6 (q22; *P* = 4.75e-07) for the japonica subpanel. These *P*-values all corresponded to *q*-values less than 0.05. The Manhattan plots of THK and NCR, chosen as examples, are represented by the three panels superimposed in Figs. 5 and 6, respectively. In a few cases, several physically close but not always adjacent markers showed the exact same level of significance. After verification, these markers appeared to be in full LD. In such cases, the extreme markers are given as an interval (Site1-Site2) in Table 3. Most of these intervals were small (on the order of 1 to 200 kb), but in at least one case (q76) on chromosome 6, the interval covered 2.5 Mb. In another case, for NR_T with the japonica panel, significant markers belonging to different chromosomes were in full LD. This situation involved 89 markers distributed across chromosomes 2, 3, 6, 7, 8 and 12. The corresponding QTL was not kept in the results table because it was not possible to unambiguously attribute it to a chromosome. Some of the QTLs were common between the whole panel and the two subpanels, more so for the indica subpanel (7 occurrences), which represents 2/3 of the whole panel accessions, than for the japonica subpanel (2 occurrences) at *P* < 1e-04. These numbers increased to 34 and 13, respectively, when decreasing the threshold to *P* <1e-03 for the significant markers (in italics in Table 3). Surprisingly, no association shared by the indica and japonica panels was detected, but half of the markers that were significant in one subpanel were monomorphic (Minor Allele Frequency (MAF) below 5 %) in the other and, were therefore, not tested (noted as nP in Table 3). In all three panels, the number of significant markers varied from trait to trait, but the range of variation was higher for the whole panel (from 1 to 14). The number of associations was greater than 5 for DEPTH (14 associations) and NCR (10 associations) in the whole panel, and for TIL (6 associations) in the japonica panel. For the remaining traits, this number was equal to or less than 5. Some of the significant markers were associated with several traits as shown in Table 4. Taking the markers that were common between panels or between traits as a single QTL, a total of 88 different sites or segments were significant at *P* < 1e-04 in this study.

**Table 3:** *P*-values of the QTLs detected as significant at *P* < 1e-04 for the whole panel, the indica and japonica subpanels

	Trait	Chr	Site1	Site2	Whole	Indica	Japonica
q1	LLGHT	3	3 555 683		7.81E-05		
q2	LLGHT	6	7 841 256	8 083 414			9.02E-05
q3	LLGHT	8	4 413 495		*3.69E-04*	9.14E-05	nP
q4	TIL	1	409 167	439 393			**2.28E-07**
q5	TIL	1	4 404 405		9.97E-05	nP	nP
q6	TIL	1	27 325 298			nP	**2.88E-05**
q7	TIL	2	10 505 253		5.33E-05	*3.96E-04*	nP
q8	TIL	3	27 657 195		1.98E-05		nP
q9	TIL	3	29 793 313		1.04E-05	*1.99E-04*	nP
q10	TIL	4	5 029 726				**3.09E-05**
q11	TIL	4	30 302 841				**4.35E-05**
q12	TIL	7	20 825 918		3.69E-05	*1.50E-04*	
q13	TIL	11	16 929 527				**3.09E-05**
q14	TIL	12	25 142 679			nP	**6.82E-05**
q15	SDW	7	18 507 925		1.44E-05	*8.22E-04*	nP
q16	SDW	8	27 413 965		5.17E-05	*3.23E-04*	nP
q17	DEPTH	1	2 231 961	2 243 573	**2.67E-07**		*8.50E-04*
q18	DEPTH	1	17 715 289		**2.68E-06**	nP	*6.06E-04*
q19	DEPTH	1	39 102 194	39 143 941	8.54E-05		
q20	DEPTH	2	6 217 128		**7.19E-06**	nP	nP
q21	DEPTH	4	20 517 263		**4.72E-06**	nP	
q22	DEPTH	6	22 826 683	22 829 858	**2.00E-05**		**4.75E-07**
q23	DEPTH	6	30 176 431		**3.20E-06**	nP	
q24	DEPTH	6	30 995 770		*2.45E-04*	nP	7.13E-05
q25	DEPTH	7	29 468 499		**4.07E-05**	nP	
q26	DEPTH	8	15 504 028	15 546 812	**4.45E-05**	nP	
q27	DEPTH	10	11 712 638		7.96E-05	nP	
q28	DEPTH	10	15 307 568		**1.43E-05**	nP	
q29	DEPTH	11	17 843 772	17 858 468			4.70E-05
q30	DEPTH	11	18 101 744				**2.10E-05**
q31	DEPTH	11	22 579 249		**6.04E-06**	nP	*7.92E-04*
q32	DEPTH	12	7 681 309		**5.64E-06**	nP	
q33	MRL	1	146 251		8.52E-05		
q34	MRL	5	18 109 976		9.30E-05	nP	
q35	MRL	6	19 870 050		4.35E-05	nP	8.04E-05
q36	NCR	1	28 579 082			nP	2.25E-05
q37	NCR	1	35 307 113	35 377 267		nP	9.02E-05
q38	NCR	2	31 652 149		2.32E-05	4.26E-05	nP
q39	NCR	3	14 543 326				5.48E-05
q8	NCR	3	27 657 195		7.07E-05		nP
q40	NCR	5	7 422 947			nP	3.93E-05
q41	NCR	6	14 126 219		**3.86E-06**	**1.06E-05**	nP
q35	NCR	6	19 870 050	20 145 257	9.49E-05		
q42	NCR	7	474 875		8.97E-05		nP
q43	NCR	11	5 272 788		**3.38E-05**	*1.12E-04*	nP
q44	NCR	11	7 927 995		**2.17E-05**	*1.66E-04*	nP
q45	NCR	11	8 972 097		**6.59E-07**	**4.40E-06**	nP
q46	NCR	11	16 559 637		**6.19E-06**	nP	nP
q47	NCR	12	1 323 261		5.07E-05	*8.00E-04*	nP
q48	NR_T	1	2 710 978	2 880 907			4.84E-05
q49	NR_T	1	10 299 033		2.11E-05	nP	
q50	NR_T	1	42 706 214		1.83E-05		
q51	NR_T	5	16 205 923	16 458 100	2.71E-05		*4.37E-04*
q52	NR_T	7	17 207 987		4.25E-05	nP	
q53	NR_T	7	22 174 085	22 175 036		5.91E-05	nP
q54	NR_T	12	27 338 111		5.54E-05	nP	nP
q55	THK	1	15 990 976	17 419 950			**2.62E-05**
q56	THK	1	19 286 868		*1.75E-04*		**4.28E-05**
q57	THK	2	35 453 974			**4.77E-07**	
q58	THK	2	35 509 414	35 510 032	**3.96E-06**		*5.41E-04*
q59	THK	3	36 156 421			3.27E-05	nP
q60	THK	6	4 649 357		*1.73E-04*	4.41E-05	nP
q61	THK	7	15 424 576				**2.46E-05**
q62	THK	8	4 544 057			8.10E-05	nP
q63	THK	11	17 111 220	17 318 688		nP	**8.14E-06**
q64	DW0020	2	25 990 556		1.10E-05	2.20E-05	nP
q65	DW0020	4	30 438 406		*9.37E-04*	3.56E-05	
q41	DW0020	6	14 126 219		4.88E-05	*1.46E-04*	nP
q45	DW0020	11	8 972 097	9 004 279	6.39E-05	*2.16E-04*	nP
q66	DW2040	2	5 015 093		6.98E-05	*2.88E-04*	nP
q67	DW2040	6	4 021 017		1.25E-05	*1.01E-04*	nP
q68	DW2040	6	17 005 559	17 036 530	6.29E-05	2.85E-05	nP
q45	DW2040	11	8 972 097	9 004 279	6.74E-05	*4.51E-04*	nP
q69	DW4060	6	4 127 010		8.00E-06	*1.03E-04*	
q70	DW4060	10	11 589 746			nP	6.75E-05
q71	DW4060	12	1 185 335		8.73E-05	*3.23E-04*	nP
q33	DWB60	1	146 251		1.94E-05		
q72	DWB60	1	2 678 840				4.85E-05
q73	DWB60	1	5 851 606		*1.87E-04*	**1.33E-06**	
q74	DWB60	2	23 550 168		1.38E-05	nP	
q75	DWB60	2	30 762 847		9.74E-05	*4.83E-04*	nP
q76	DWB60	6	14 008 461	16 515 631	6.55E-05		
q77	DWB60	6	16 798 522		3.49E-05		
q73	DRW	1	5 851 606		*2.56E-04*	2.82E-05	
q78	DRW	2	14 366 103				9.79E-05
q69	DRW	6	4 127 010		2.92E-05	9.76E-05	
q79	DRW	6	16 912 708		1.44E-05		
q70	DRW	10	11 589 746		*8.85E-04*	nP	2.31E-05
q69	RDW	6	4 127 010		3.25E-05	*4.37E-04*	
q44	RDW	6	14 126 219		9.79E-05	*3.36E-04*	nP
q45	RDW	11	8 972 097	9 004 279	1.48E-05	9.22E-05	nP
q15	PDW	7	18 507 925		2.39E-05	*9.05E-04*	nP
q45	PDW	11	8 972 097	9 004 279	4.97E-05	*3.07E-04*	nP
q80	SRP	4	13 232 104		4.12E-05	nP	nP
q81	SRP	6	1 901 540	1 930 717	3.11E-05		
q82	SRP	6	21 716 314	21 716 317		6.59E-05	
q83	DRP	1	2 173 691		2.64E-05		*2.95E-04*
q84	DRP	1	6 404 307		*8.46E-04*	5.93E-05	
q85	DRP	4	4 170 196			6.71E-05	
q82	DRP	6	21 716 314			6.28E-05	
q86	R_S	6	4 601 154		8.15E-06		*6.90E-04*
q87	R_S	6	9 185 455		8.91E-05	*3.32E-04*	
q88	R_S	6	24 668 182		*2.20E-04*	nP	8.14E-05

The *P*-value of the test in the three panels up to *P* = 1e-03 is given in italics. In bold, QTLs with *q*-values < 0.05

*Chr* chromosome, *nP* not polymorphic in the sub-panel (monomorphic or MAF < 5 %), *LLGTH* longest leaf length, *TIL* number of tillers, *SDW* shoot dry weight, *DEPTH* deepest point reached by roots, *MRL* maximum root length, *NCR* number of crown roots, *NR_T* number of crown root per tiller, *THK* root thickness, *DW0020* root mass in the 00–20 cm segment, *DW2040* root mass in the 20–40 cm segment; *DW4060* root mass in the 40–60 cm segment, *DWB60* root mass below 60 cm, *DRW* deep root mass (<40 cm) weight, *RDW* root dry weight, *PDW* plant dry weight, *SRP* shallow root proportion (0–20 cm), *DRP* deep root proportion (<40 cm), *R_S* root to shoot ratio

**Table 4:** QTLs common across traits for the three panels

QTLs	Chr	Position	Nb ass.	LLGHT	TIL	DEPTH	MRL	NCR	NR_T	THK	DW0020	DW2040	DW4060	DWB60	DRW	RDW	SDW	PDW	SRP	DRP	R_S
A. Whole panel																					
q33	1	146 251	3				*							*						(*)	
q83	1	2 173 691	2				(*)													*	
q66	2	5 015 093	2									*				(*)					
q8	3	27 657 195	2		*			*													
q67	6	4 021 017	4									*	(*)		(*)	(*)					
q24	6	4 127 010	5								(*)	(*)	**		*	*					
q76	6	14 008 461	6					**			*			*	(*)	*		(*)			
q79	6	16 912 708	3										(*)		*	(*)					
q15	7	18 507 925	3												(*)		*	*			
q16	8	27 413 965	2														*	(*)			
q43	11	5 272 788	2					*			(*)										
q45	11	8 972 097	9		(*)			***			*	*			(*)	*	(*)	*			*
q31	11	22 579 249	2			**	(*)														
q32	12	7 681 309	2			**	(*)														
B. Indica subpanel																					
q73	1	5 851 606	3			(*)								**	*						
q84	1	6 404 307	2								*				(*)					*	
q65	4	30 438 406	2								*									(*)	
q69	6	4 127 010	3										(*)		*	(*)					
q41	6	14 126 219	3					*			(*)					(*)					
q68	6	17 005 559	3									*	(*)		(*)						
q82	6	21 716 314	2																*	*	
q3	8	4 413 495	2	*						(*)											
q45	11	8 972 097	6					**			(*)	(*)			(*)	*		(*)			
C. Japonicasubpanel																					
q72	1	2 678 840	2											*						(*)	
q48	1	2 710 978	2						*												
q40	5	7 422 947	2		(*)			*													
q70	10	11 589 746	3										*		*	(*)					

A whole panel; B indica subpanel; C japonica subpanel

*Chr* chromosome, *Nb ass.* number of associations, *LLGTH* longest leaf length, *TIL* number of tillers, *SDW* shoot dry weight, *DEPTH* deepest point reached by roots, *MRL* maximum root length, *NCR* number of crown roots, *NR_T* number of crown root per tiller, *THK* root thickness, *DW0020* root mass in the 00–20 cm segment, *DW2040* root mass in the 20–40 cm segment, *DW4060* root mass in the 40–60 cm segment, *DWB60* root mass below 60 cm, *DRW* deep root mass (<40 cm) weight, *RDW* root dry weight, *PDW* plant dry weight, *SRP* shallow root proportion (0–20 cm), *DRP* deep root proportion (<40 cm), *R_S* root to shoot ratio

(*): *P* < 1e-03; *: *P* < 1e-04; **: *P* < 1e-05; ***: *P* < 1e-06

### Function of genes that were linked to significant markers

Among the 88 different sites identified, 33 were in genes with predicted functions. Given the level of LD in the panel, the genes that were within an interval of +/-25 kb on both sides of the significant markers were also surveyed using the query tools of OrygenesDB [23] to retrieve 889 additional genes, of which 407 had predicted functions (Additional file 6: Table S5). This list of QTL-associated genes was first compared to the list of approximately 200 genes that were recorded in EURoot database [24] that are known, mostly via mutant analysis, to play roles in rice root architecture, root development or water and nutrient transport. No correspondence was found except for *PLASMA MEMBRANE INTRINSIC PROTEIN 2;1* (*OsPIP2;1*) gene located 16 kb from q61 on chromosome 7, which was significant for THK in the japonica panel (Table 5). The list of QTL-associated genes was similarly compared with a list of genes that are specifically expressed during crown root formation or development [25, 26]. Eleven of the QTL-associated genes corresponded to genes that are specifically expressed in different zones of the crown root such as the root cap, the lateral root differentiation zone and the mature zone (Table 5). Most of these genes had a predicted biochemical function, but no precise information could be found regarding their biological functions. Selecting only those genes associated with root trait QTLs, the literature was then scanned to determine whether information about their biological function or that of their predicted *Arabidopsis* ortholog(s) was available. This approach revealed 13 additional interesting candidate genes, which are also listed in Table 5.

**Table 5:** List of candidate genes close to the significant markers determined based on function or expression pattern

QTL ID	Chr	Position of QTL	Trait(s)	Gene in rice	Gene in *Arabidopsis*	Function	References
q48	1	2710978-2880907	NR_T	Os01g05820	no	Specifically expressed in lateral root initiation zone	[25]
q48	1	2710978-2880907	NR_T	Os01g06010	no	Specifically expressed in lateral root initiation zone	[25]
q48	1	2710978-2880907	NR_T	Os01g06060	no	Specifically expressed in root cap	[25]
q73	1	5851606	DWB60, DRW	Os01g10900	At04g18640	*Arabidopsis MRH1*, root hair development	[44]
q73	1	5851606	DWB60, DRW	Os01g11010	no	Specifically expressed in lateral root initiation zone	[25]
q84	1	6404307	DRP	Os01g11860	no	Specifically expressed in root cap	[25]
q49	1	10299033	NR_T	Os01g18360	no	*OsIAA4*, crown roots formation in response to auxin	[34]
q36	1	28579082	NCR	Os01g49690	At01g50370	*Arabidopsis FYPP1*, post transductional, *PIN* protein regulation	[32]
q50	1	42706214	NR_T	Os01g73700	no	Specifically expressed in root cap	[25]
q50	1	42706214	NR_T	Os01g73720	no	Specifically expressed in root cap	[25]
q66	2	5015093	DW2040	Os02g09760	no	Specifically expressed in root cap	[25]
q64	2	25990556	DW0020	Os02g43 120	no	Specifically expressed in root cap	[25]
q74	2	30762847	DWB60	Os02g50372	no	Candidate gene for a root mass at depth QTL	[45]
q38	2	31652149	NCR	Os02g51710	At04g39730	*Arabidopsis PLAT1*, regulation of lateral root development	[41]
q59	3	35166421	THK	Os03g63970	At05g51810	*OsGA20ox1*, underlies a QTLs for early vigor	[47]
q51	5	16205923-16458100	NR_T	Os05g27920	At05g58440	*Arabidopsis SNX2*, endocellular transport of PIN2	[33]
q60	6	4 649 357	THK	Os06g09280	At01g26370	*Arabidopsis RID1*, root development	[46]
q76	6	15 865 171	DWB60	Os06g27980	At03g05390	Specifically expressed in root cap	[25]
q42	7	474 875	NCR	Os07g01820	no	*OsMADS15*, crown roots development	[39, 40]
q61	7	15 424 576	THK	Os07g26740	no	*OsRR7*, cytokinin signaling	[49, 57]
q61	7	15 424 576	THK	Os07g26740	no	Specifically expressed in lateral root initiation zone	[25]
q53	7	22174085-22175036	NR_T	Os07g37010	At04g03270	*Arabidopsis CYCLIN D6;1* lateral root initiation	[38]
q71	12	1 185 335	DW4060	Os12g03110	no	*Arabidopsis MIF1*, root development	[43]
q71	12	1 185 335	DW4060	Os12g03150	At01g08810	*Arabidopsis MYB60*, root development	[42]

## Discussion and conclusions

We have phenotyped the root traits of a panel of 182 Vietnamese varieties in a soil-based phenotyping system to analyze the genetic control of root architecture.

The phenotypic variation of the panel was analyzed at the light of its genetic structure for GWAS purpose. The japonica subpanel showed on average poor performance, with lower mean values than the indica subpanel for deep root traits and biomasses. However, the analysis per sub-population shows that the overall subpanel performance masked important differences between subpopulations. These differences seem to be mostly related to ecosystem adaptation and risks of drought, as also shown by Lafitte et al. [26] with a sample of varieties originating from across Asia. This is true for the japonica subpanel, for which subpopulations J1 and J3, with the deepest and thickest roots, include mostly upland rice varieties, while subpopulations J2 and J4, with thin and shallow roots, correspond to irrigated and mangrove rice varieties, respectively [22]. More surprisingly, the same is also true for the indica subpanel: the two best subpopulations (I3 and I6) are almost exclusively composed of upland accessions (I3) and from a mixture of upland and rainfed lowland accessions (I6), whereas the two subpopulations (I1 and I4) with the poorest performance originated from irrigated ecosystems [22]. The indica types from group I3 constitute interesting donors of deep and thick roots that may be easier to use as parents in crosses with other indica vaccessions than the upland japonica accessions, by reducing the risks of F_1_ inter subspecies sterility. The global organization of root variability in this panel highlights the need to control population structure and, because the panel phenotypic differences partly overlapped with the genetic structure for some of the traits, to perform individual analyses of each subpanel rather than only performing an analysis of the whole panel.

In this study, we detected QTLs for all the examined traits. Their number is generally limited, but some QTLs show both good *P*-values (*P* < 1e-06) and *q*-values (*q* < 0.05) and seem to merit further research, particularly the QTL for THK on chromosome 2 and for NCR on chromosome 11.

We found some significant markers located on different chromosomes but in full LD. In such situation, it is impossible to know where the functional mutation is located. This situation of full LD between markers far apart or on different chromosomes is probably much more frequent than was observed in our study but is difficult to detect for non-significant markers and generally goes unnoticed. The risk of such situations is likely higher when the panel size is low, as is the case here for the japonica subpanel.

Generally, the markers that were significant for a given trait in the whole panel were also significant for the same trait in the indica or the japonica panel, albeit with lower levels of significance. However, we found ten markers significant in the whole panel but not significant in any of the two subpanels. We did not find any markers associated with the same traits in both the indica and japonica subpanels. These differences in associations detected between panels may be partly due to the limited size of the subpanels, notably of the japonica subpanel, which limits our detection power. A panel size below 100 accessions is considered as suboptimal for association detection, notably for low MAF markers [15]. However, as shown in the example of Zhao et al. [27] with subpanel sizes (57 to 97 accessions) similar to that of our japonica subpanel, significant associations can still be detected when the marker effects are large. The differences in associations detected between panels may also partly result from the large variation in allelic frequencies in the different panels observed for many markers. These variations themselves result from the strong bipolar organization of the genetic diversity in rice due to the independent domestications of indica and japonica subspecies followed by limited introgressions from one subspecies to the other, mainly in areas of low divergence [28, 29]. This second possibility is supported by the reduction of the set of polymorphic markers (MAF greater than 5 %) from 21,623 in the whole panel to 13,814 in the indica subpanel and 8821 in the japonica subpanel. Many of the markers that were polymorphic in one subpanel were considered monomorphic (MAF below 5 %) in the other. Only 20.1 % of the 21,623 initial markers were polymorphic in both subpanels, while 15.4 % were monomorphic in both but with different alleles (markers discriminating indica from japonica accessions).

When comparing the significant associations identified in this study with those identified by [17] for similar root traits in a japonica panel with no common accessions except the checks, we found one case of co-localization (*d* <50 bp). A marker that was significantly associated with NR_T in our study (q50 at position 42.706 Mb on chr 1) was associated with R_S in their study. These authors did not measure NR_T. However, we cannot exclude that this co-localization occurred by chance because the same marker is very far from the threshold of significance for R_S in any panel in our study.

We assessed the function of the genes in which or near which significant markers were located. *OsPIP2;1* (*Os07g26690*), at 16 kb of q61, is more specifically expressed in the root exodermis and up-regulated specifically in roots of upland varieties after osmotic stress treatment [30, 31]. However, the three markers that were found between the QTL and the gene showed a decreasing level of significance when approaching the gene, and it is difficult to establish a link between the function of this protein and the phenotype (THK) that is associated with the related QTL.

The literature searches for the biological functions of the QTL-associated genes and their predicted *Arabidopsis* ortholog(s) led to interesting candidates. For example, crown root initiation and development in rice are known to involve local auxin flux regulation by the relocation of PIN-FORMED (PIN) auxin efflux proteins, resulting in the activation by auxin of the gene encoding the LATERAL ORGAN BOUNDARY DOMAIN (LBD) transcription factor CROWN ROOT LESS 1 (CRL1) [11, 13, 14, 32]. Some genes that are involved in this regulatory pathway were included in the confidence interval of QTLs that were related to NCR or NR_T. *Os01g49690* (NCR, q36) is an ortholog of *Arabidopsis PHYTOCHROME-ASSOCIATED SERINE/THREONINE PROTEINPHOSPHATASE1* (*FYPP1*). The *Fypp1*; *Fypp2* double mutant is characterized by an elevation of a phosphorylated PIN protein that results in a basal-to-apical subcellular PIN accumulation, an increase in root basipetal auxin transport, and a phenotype showing shorter roots and less lateral root formation [33]. *SORTING NEXIN2* (*SNX2a)*, the *Arabidopsis* ortholog of *Os05g27920* (NRT_T, q51), shares partial functional redundancy with *SNX1*, which is involved in the endocellular transport of PIN2 via the formation of SNX1-containing endosomes [34]. AUXIN/INDOLE-3-ACETIC ACID (IAA) are negative regulators of the auxin response and are involved in *CRL1*gene expression regulation by auxin [32]. The over-expression of *OsIAA4* (*Os01g18360*, NR_T, q49) results in a reduction of the number of crown roots that form after auxin treatment, suggesting that this gene may be involved in the auxin signaling pathway that controls the formation of crown roots [35]. CRL1 regulates genes involved in meristem formation and patterning, such as *SCARECROW* (*SCR*), which acts together with *SHORTROOT* (*SHR*) to control the division of the cortex-endodermis initial cell in *Arabidopsis* or with *QUIESCENT CENTER HOMEOBOX* (*QHB*), which is an ortholog of the *WUSCHEL*-related *WOX5* gene, to contribute to the quiescent center and root stem cell specification and maintenance [36–38]. Finally, *Os07g37010* (NR_T, q53) is the ortholog of *Arabidopsis CYCLIN D6;1* that was identified as a direct target of *SHR* and *SCR* and is involved in the cell division events leading to root meristem formation and function [39]. All of these genes may be involved upstream or downstream of CRL1 in the regulation of crown root formation. *OsMADS15* (*Os07g01820*, NCR, q42) over-expressing plants display alterations in development, including the over development of crown roots associated with enhanced expression of the transcription factor WOX11, another gene that controls the formation and development of crown roots through a CRL1-independent pathway [40, 41]. *Os02g51710* is an ortholog of *Arabidopsis POLYCYSTIN-1, LIPOXYGENASE, ALPHA-TOXIN AND TRIACYLGLYCEROL LIPASE 1* (*PLAT1*). *PLAT1* is induced by abiotic stress and positively regulates lateral root development [42].

Some genes whose function is related to root development and that are close to QTLs for deep-root biomass have been also identified. *Os12g03150* (DW4060, q71) is an ortholog of *Arabidopsis AtMYB60*, which controls stomatal closure and stimulates root development in response to drought [43]. *Os12g03110* (DW4060, q71) is the ortholog of *MINI ZINC FINGER 1* (*MIF1*), which is a negative regulator of plant development, including root development [44]. *Os01g10900* (DWB60, RDW, q73) is the ortholog of *Arabidopsis MORPHOGENESIS OF ROOT HAIR 1* (*MRH1*), which regulates root-hair development [45]. Finally, *Os02g50372* (DWB60, q74), is an expressed protein of unknown function but was previously reported as a candidate gene underlying a QTL that is related to root mass at depth, based on expression studies [46].

Concerning root thickness, *Os06g09280* (THK, q60) is an ortholog of *ROOT INITIATION DEFECTIVE 1* (*RID1*). *RID1* is involved in different developmental processes, such as meristem maintenance, and leaf and root morphogenesis. In roots, *RID1* contributes to the proper expression of *SCR* and *WOX5*, two key genes involved in meristem shaping and root- tissue patterning [47]. *Os03g63970* (THK, q59) corresponds to *GIBBERELIC ACID 20 OXIDASE 1* (*OsGA20ox1*), which is a gene underlying a previously identified QTL for vigor at early developmental stages [48]. In *Arabidopsis*, GA, together with *SCR* and *SHR*, plays a key role in regulating the differentiation of supernumerary cortex cell layers in roots [49]; thus, the function of *OsGA20ox1* should be further investigated. Similarly, cytokinins play a key role with auxin in root vascular patterning [50]. *Os07g26740* (THK, q61), which encodes the *RESPONSE REGULATOR 7* (*OsRR7*), may be involved in this process.

Several genes were identified whose reported biological function is consistent with the associated phenotype, the rice gene or its predicted *Arabidopsis* ortholog (Table 5). Knock-down or gain-of-function mutants of these genes should be generated to further investigate their function in the related phenotypes. Nevertheless, the fact that two genes are predicted to be orthologs in rice and *Arabidopsis* based on sequence homology does not guarantee that their function is conserved between the two species. In addition, several genes supporting QTLs initially had unknown functions [51, 52]. Therefore, to further validate these QTLs, it will be interesting to conduct an exhaustive differential expression study between contrasting varieties for the related phenotype for all genes included in the confidence interval of a given QTL. This approach has often been used as a criterion to restrict the number of candidate genes underlying a QTL independently of their putative function [46, 48, 53]. Another way to validate these QTLs, which were detected on a statistical basis, is to develop mapping populations segregating for the QTL. Although time consuming, this approach is a necessary step towards the positional cloning of the QTL. Marker haplotypes were established in the regions of the QTLs for NCR on chromosome 11 and THK on chromosome 2, which are the two most significant QTLs. Accessions of the panel with contrasting haplotypes will be used in a future study to make crosses and develop mapping populations in an indica background for NCR and in a japonica background for THK.

## Methods

### Materials

The material that was used in the experiment was composed of a panel of 197 accessions from Vietnam and 3 controls (Nipponbare, IR64 and Azucena). The seeds from the Vietnamese accessions came from the Plant Resource Center, Hanoi, Vietnam. These accessions were mostly traditional lines originating from various regions of Vietnam. The accession name, ID number, province of origin, ecosystem of origin, varietal group (indica (I), japonica (J) or intermediate (m)) and subpopulation within the varietal group (I1 to I6 for the indica accessions and J1 to J4 for the japonica accessions), when known, are listed in Additional file 1: Table S1. Other characteristics of the accessions can be found in Phung et al. [22]. The accessions were seed increased for one generation under field conditions, and one plant representative of the plot was selected for DNA extraction and further phenotyping.

### Methods

#### Phenotyping experiment

The experiment was conducted at Van Giang Agricultural Station (latitude 20°39′ N, longitude 106°3′ W) near Hanoi, Vietnam, in August-September 2012. The experimental design was an alpha-lattice with 3 replicates. A block factor (10) was introduced to control for possible environmental variations within replicates. A block included 20 accessions. The experimental unit was composed of one plant.

#### Conditions of the experiment

The plants were grown under screenhouse conditions in well-drained black plastic bags, 80 cm long and 16 cm wide, that were filled with fine river sand and supplemented with fertilizer (5/10/3 NPK at the rate of 20 kg/m^3^ of sand). The seeds were sown on watered filter paper, incubated at 28 °C for 3 days at obscurity and transplanted into bags (one seed per bag). Three accessions did not germinate. The plants were watered four times a day (6 am, 10 am, 2 pm, and 6 pm) except during rain. Fertilizer (2.5 g 15/15/15 N/P/K per plant,) was added three weeks after transplanting following solubilization in water. The root system was collected 45 days after sowing.

For each plant, the length of the longest leaf was measured (LLGTH). The number of tillers per plant (TIL) was counted. The shoot part was dried for 3 days at 72 °C in an oven and weighed (SDW). The deepest point that was reached by the roots in the bag was measured (DEPTH). Then, the roots were carefully washed. The maximum root length (MRL) was measured. The number of crown roots (NCR) below the tillering plateau was counted, and the number of crown roots per tiller (NR_T) was computed as NCR/TIL. The root thickness (THK) was measured using a micrometer on 5 roots per plant, 2 cm below the tillering plateau, and averaged. The root system was divided into 4 segments from the top to the bottom (0 to 20 cm (DW0020), 20 to 40 cm (DW2040), 40 to 60 cm (DW4060) and below 60 cm (DWB60)) and the segments were oven-dried for 3 days at 72 °C. The root dry weight (RDW) and deep root weight (DRW) were computed as the sum of the four segments and the sum of the two deeper segments (DW4060 + DWB60), respectively. The shallow root weight proportion (SRP) was computed as DW0020*100/RDW. The deep root weight proportion (DRP) was computed as DRW*100/RDW. The root-to-shoot ratio (R_S) was computed as SDW*100/RDW.

To represent the measured phenotypic traits on a synthetic figure, a tracing software named “RASTA” (for Root And Shoot TrAcing) was developed using the PHP hypertext preprocessor (PHP, The PHP Group, http://www.php.net) coding language and a drawing set of instructions from the Graphics Draw Graphics Library (http://www.libgd.org). The software is available from the corresponding author. The shoot and root parts of each accession were drawn as two polygons whose dimensions were proportional to the considered traits (see Additional file 3: Figure S1 for detailed explanations).

#### Genotyping by Sequencing (GBS)

GBS data were available for 182 Vietnamese accessions of the 197 that germinated, and for the three controls. The GBS method and the selection of the resulting SNP markers are described in detail in [22]. Briefly, the genome complexity reduction was done using PstI/TaqI restriction digest, a combination of enzymes enabling a good sequencing depth, and was followed by Illumina short read sequencing. Markers that had no position on the Nipponbare sequence or that had more than 20 % missing data were discarded. Because of the sensitivity of GWAS to the presence of unbalanced genotypic classes markers that had a minor allele frequency (MAF) of less than 5 % were also discarded. The remaining missing data (5.2 % in the whole date set) were imputed using Beagle v3.3.2 [54]. The final data set was composed of 21,623 markers without missing data. Indica (115 accessions) and japonica (64 accessions) subpanels were also individualized and contained 13,814 and 8821 markers, respectively, that were polymorphic and with an MAF of less than 5 % in their specific subpanel.

#### Statistical analyses

An analysis of variance (ANOVA) was performed considering the variety, replicate and block effects as fixed. The fixed effects were tested using SAS 9.2. (SAS Institute, Cary NC, USA) and least square variety means were computed to adjust for imbalance of the block effects. The phenotypic correlations between traits were computed using these adjusted means. To analyze the organization of the phenotypic variability, a Principal Component Analysis (PCA) was undertaken on the adjusted means of all of the traits. To further analyze the relationships between phenotypic variability and genetic structure, which can deeply affect the results of GWAS, three independent ANOVAs were performed on the adjusted means of each trait considering a group effect as the main factor within the whole panel, and considering a subpopulation effect as the main factor within each of the two subpanels, and using a Newman and Keuls test to detect differences between group means or between subpopulation means. For each trait-by-panel combination, the percentage of phenotypic variance that was explained by the structure was computed by a regression of the phenotype on the percentages of admixtures that were obtained by Phung et al. [22] for all accessions. These analyses were performed using SAS v9.2, except for the PCA, for which XLStat [55] was used.

#### Association mapping

We performed successive association analyses on the whole panel (182 accessions * 21,623 markers), and on the indica (115 accessions * 13,814 markers) and japonica (64 accessions * 8821 markers) subpanels using Tassel v3 [56]. Three accessions of the whole panel classified as admixed (% of admixture below 75 % in any subpanel [22]) were excluded from the subpanels. For all of the traits, we used a mixed model with a structure matrix that was considered as a fixed effect and a kinship matrix considered as a random effect as covariates to control the false-positive rate. We chose the options of no compression and re-evaluation of variance components for each marker. The structure matrices of the three panels were determined by running a PCA on their respective marker data sets. For the three panels, the first six PCA axes were retained, and the scores of the accessions on these six axes were used as the structure matrix. The kinship matrices were established using the pairwise Identity by State (IBS) method proposed by Tassel. Quantile-quantile plots (QQ plots) were drawn using Tassel. QQ plots enable to graphically evaluate the number of false positives observed with the chosen model, based on deviations from the uniform law. The inflation factor lambda was computed to quantitatively assess the extent of these deviations using the “estlambda” function from the R GenABEL package. The expected value of lambda is 1 for no inflation situations. We used a threshold of 1e-04 to declare an association significant. To determine this threshold, the effective number of independent tests (Meff) was first computed using the R SimpleM package and was used as the denominator in the Bonferroni correction formula as proposed by Gao [57]. The method is said to give close approximation to a permutation threshold. Meff were 8545, 5649, and 2310 for the whole panel, the indica and the japonica subpanels, respectively, corresponding to *P* values of approximately 5e-06, 1e-05, and 5e-05. Since we wanted to be able to make comparisons across populations and across traits, we chose the less stringent common *P* value of 1e-04. Then, we computed the *q*-value corresponding to each *P*-value for all of the traits in all three panels using the R Q-value package v1.0 [58] as a measure of the false-discovery rate. The associations that were significant at a *q*-value < 0.05 were represented in bold in Table 3. The Manhattan plots that graphically represent the significance of all markers were drawn using Tassel.

## Availability of supporting data and material

The GBS dataset (hapmap format) supporting the results of this article has been deposited as a downloadable Excel file in TropGeneDB: http://tropgenedb.cirad.fr/tropgene/JSP/interface.jsp?module=RICE tab “studies”, study type “genotype”, study “Vietnamese panel - GBS data”.

The seeds of the accessions are available from the Plant Resource Center, Hanoi, Vietnam.

## Additional files

Additional file 1: Table S1. List of the 200 accessions used in the phenotyping experiments. (DOCX 56 kb) Additional file 2: Table S2. Mean, standard deviation, range, and CV of the whole panel for all traits measured. (DOCX 31 kb) Additional file 3: Figure S1. Graphical representation of the panel. (PPTX 418 kb) Additional file 4: Table S3. Mean comparisons between groups in the whole panel and subpopulations in each subpanel and percentage of variance explained by the structure (% var). (DOCX 48 kb) Additional file 5: Table S4. Pearson correlation coefficients between traits in the whole panel (below the diagonal). Probabilities above the diagonal (in bold, significant at *P* < 0.05). W = whole panel; I = indica panel; J = japonica panel. (DOCX 43 kb) Additional file 6: Table S5. List of genes with function located nearby the QTLs. (PDF 146 kb)

## Abbreviations

ANOVA: analysis of variance
CRL1: crown root less 1
DEPTH: deepest point reached by roots
DRP: deep root proportion (<40 cm)
DRW: deep root mass (<40 cm) weight
DW0020: root mass in the 00–20 cm segment
DW2040: root mass in the 20–40 cm segment
DW4060: root mass in the 40–60 cm segment
DWB60: root mass below 60 cm
FYPP: phytochrome-associated serine/threonine proteinphosphatase
GA20ox: gibberelic acid 20 oxidase
GBS: genotyping by sequencing
GWAS: genome-wide association studies
IAA: auxin/indole-3-acetic acid
LBD: lateral organ boundary domain
LD: linkage disequilibrium
LLGTH: longest leaf length
MAF: minor allele frequency
MRH: morphogenesis of root hair
MRL: maximum root length
NCR: number of crown roots
NR_T: number of crown root per tiller
PCA: principal components analysis
PDW: plant dry weight
PHP: hypertext preprocessor
PIN: pin-formed
PIP: plasma membrane intrinsic protein
PLAT: polycystin-1, lipoxygenase, alpha-toxin and triacylglycerol lipase
QHB: quiescent center homeobox
QQ: quantile-quantile
QTLs: quantitative trait loci
MIF: mini zinc finger
RDW: root dry weight
RID: root initiation defective
RR: response regulator
R_S: root to shoot ratio
SCR: scarecrow
SDW: shoot dry weight
SHR: shortroot
SNP: single nucleotide polymorphism
SNX: sorting nexin
SRP: shallow root proportion (0–20 cm)
THK: root thickness
TIL: number of tillers
WOX: WUSCHEL-related
