# Supplementary material for: Genome-wide association mapping for root traits in a panel of rice accessions from Vietnam
Source: BMC Plant Biol. 2016 Mar 10;16:64. doi: 10.1186/s12870-016-0747-y (PMC4785749; doi:10.1186/s12870-016-0747-y)
Supplement: Additional file 1: Table S1. — List of the 200 accessions used in the phenotyping experiments. (DOCX 56 kb) [file 12870_2016_747_MOESM1_ESM.docx]

Table S1: List of the 200 accessions used in the phenotyping experiments

| ID | Name | Genebank number | Province | Type | Ecosyst. | Group | Sub |
| --- | --- | --- | --- | --- | --- | --- | --- |
|  |  |  |  |  |  |  | pop |
| AZ | AZUCENA | IRGC_328 | na | IMP | UP | J | J |
| G1 | TEP HAI PHONG | VNPRC_11 | HAI PHONG | TRAD | na | I | Im |
| G10 | TAM SON NAM DINH | VNPRC_216 | NAM DINH | TRAD | na | I | I4 |
| G100 | KHAU QUAI DANG 2 | VNPRC_6969 | TUYEN QUANG | TRAD | UP | I | J1 |
| G101 | DIEO KBIN | VNPRC_7295 | TAY NGUYEN | TRAD | na | J | J1 |
| G102 | TZO KOH DANG 2 | VNPRC_7303 | THUA THIEN HUE | TRAD | na | I | Im |
| G103 | CU PUA DANG 1 | VNPRC_7304 | THUA THIEN HUE | TRAD | na | J | Jm |
| G104 | CU PUA DANG 2 | VNPRC_7305 | THUA THIEN HUE | TRAD | na | I | Im |
| G105 | NEP THAI LAN | VNPRC_7312 | HA GIANG | TRAD | na | I | I3 |
| G106 | NEP HAI HAU | VNPRC_7316 | NINH BINH | TRAD | IR | J | J2 |
| G107 | NEP THAI BINH LUN | VNPRC_7317 | NINH BINH | TRAD | IR | J | J2 |
| G108 | TAM AP BE | VNPRC_7318 | NINH BINH | TRAD | IR | m | m |
| G109 | MANH GIE | VNPRC_7349 | QUANG BINH | TRAD | UP | I | Im |
| G11 | TAM TRON HAI DUONG | VNPRC_219 | HAI DUONG | TRAD | na | I | I4 |
| G110 | RAN TRANG | VNPRC_7823 | BINH THUAN | TRAD | UP | I | Im |
| G111 | NEP RAY | VNPRC_7824 | BINH THUAN | TRAD | UP | I | Im |
| G113 | NANG THIET | VNPRC_7827 | VUNG TAU | TRAD | IR | I | I2 |
| G115 | KOI LOI | VNPRC_7910 | na | TRAD | na | I | Im |
| G116 | KOI PU | VNPRC_7913 | na | TRAD | na | I | na |
| G117 | KHAO SANG | VNPRC_7930 | QUANG TRI | TRAD | UP | J | Jm |
| G118 | L03 | VNPRC_9175 | na | IMP | na | I | na |
| G119 | L26 | VNPRC_9198 | na | IMP | na | I | Im |
| G12 | TAM CAO VINH PHUC | VNPRC_226 | VINH PHUC | TRAD | na | I | I4 |
| G120 | BAY THANH | VNPRC_9219 | CA MAU | TRAD | na | I | I2 |
| G121 | CA RO | VNPRC_9246 | TAY NINH | TRAD | IR | I | Im |
| G124 | NEP DEN | VNPRC_9355 | QUANG NINH | TRAD | IR | J | J2 |
| G125 | NEP NUONG | VNPRC_9356 | QUANG NINH | TRAD | na | I | Im |
| G126 | KHAU DAM DOI | VNPRC_9466 | NGHE AN | TRAD | UP | J | J1 |
| G128 | KHAU DAM | VNPRC_9476 | NGHE AN | TRAD | UP | J | J1 |
| G129 | LC93-2 | VNPRC_9507 | KHANH HOA | TRAD | UP | I | Im |
| G13 | TAM DEN HAI PHONG | VNPRC_253 | HAI PHONG | TRAD | na | I | na |
| G130 | LUA DA BO | VNPRC_9509 | KHANH HOA | TRAD | UP | J | J3 |
| G131 | PADAI LONG KHANH | VNPRC_9517 | KHANH HOA | TRAD | UP | J | J3 |
| G132 | PADAI TLIG JUG | VNPRC_9520 | KHANH HOA | TRAD | UP | I | Im |
| G133 | A 330 | VNPRC_9524 | KHANH HOA | IMP | IR | I | I1 |
| G134 | PADAI CALOC | VNPRC_9530 | KHANH HOA | TRAD | UP | J | Jm |
| G135 | PADAI DLUC | VNPRC_9532 | KHANH HOA | TRAD | UP | J | na |
| G136 | PHUOC LONG | VNPRC_9541 | KHANH HOA | TRAD | UP | I | Im |
| G137 | NEP NGHE | VNPRC_9554 | BEN TRE | TRAD | RL | I | na |
| G138 | NANG QUAT | VNPRC_9563 | BEN TRE | TRAD | RL | I | Im |
| G139 | LUA NANG DEN | VNPRC_9568 | BEN TRE | TRAD | RL | I | I2 |
| G14 | TAM NHO BAC NINH | VNPRC_318 | BAC NINH | TRAD | na | I | I4 |
| G140 | LUA BAY DANH | VNPRC_9570 | BEN TRE | TRAD | RL | I | I2 |
| G141 | LUA NANG NIEU CHUM | VNPRC_9573 | BEN TRE | TRAD | RL | I | I2 |
| G142 | LUA THANH TRA | VNPRC_9574 | BEN TRE | TRAD | RL | I | I2 |
| G143 | NEP TROI CHO | VNPRC_9576 | BEN TRE | TRAD | RL | I | I2 |
| G144 | LUA MUA DIA PHUONG | VNPRC_9578 | BEN TRE | TRAD | RL | I | I2 |
| G145 | BA RIA | VNPRC_9580 | BEN TRE | TRAD | RL | J | J1 |
| G146 | NANG LOAN HAT TRON | VNPRC_9584 | BEN TRE | TRAD | RL | I | I2 |
| G147 | LUA NANG LOAN HAT DAI | VNPRC_9585 | BEN TRE | TRAD | IR | I | I2 |
| G148 | TRANG TEP | VNPRC_9588 | BEN TRE | TRAD | RL | I | na |
| G150 | NEP DIA PHUONG | VNPRC_9595 | BEN TRE | TRAD | IR | I | I2 |
| G152 | LOC SOM | VNPRC_9871 | BAC GIANG | TRAD | na | J | J1 |
| G153 | TE NUONG | VNPRC_9874 | THANH HOA | TRAD | UP | I | I3 |
| G154 | NEP THOM | VNPRC_9878 | HA TAY | TRAD | IR | J | J2 |
| G155 | KHAU PE LANH | VNPRC_9908 | SON LA | TRAD | UP | I | I3 |
| G156 | LUA K | VNPRC_9967 | na | TRAD | na | I | Im |
| G157 | SO CRIOONG | VNPRC_9984 | SEKONG | TRAD | na | J | Jm |
| G158 | VA TAI ANA ACU | VNPRC_12049 | NINH THUAN | TRAD | UP | J | J3 |
| G16 | NEP VAN RUONG HOA BINH | VNPRC_384 | HOA BINH | TRAD | na | J | J2 |
| G160 | JASMINE | VNPRC_12059 | AN GIANG | IMP | UP | I | Im |
| G161 | BN1 | VNPRC_12066 | AN GIANG | IMP | IR | I | Im |
| G162 | NEANG CON | VNPRC_12068 | AN GIANG | TRAD | UP | I | I2 |
| G163 | CA CHOCH CHAP | VNPRC_12071 | AN GIANG | TRAD | UP | I | Im |
| G165 | GIONG 90 NGAY | VNPRC_12083 | KIEN GIANG | TRAD | RL | I | I1 |
| G166 | CHIN TEO | VNPRC_12086 | KIEN GIANG | TRAD | na | I | I2 |
| G167 | THAN NONG MUA | VNPRC_12088 | KIEN GIANG | TRAD | RL | I | I2 |
| G168 | OM1490 | VNPRC_12101 | KIEN GIANG | IMP | IR | I | I1 |
| G169 | JASMINE 95 | VNPRC_12102 | KIEN GIANG | IMP | IR | I | I1 |
| G17 | NEP GA GAY HAI DUONG | VNPRC_394 | HAI DUONG | TRAD | na | I | Im |
| G170 | OM 504 JAPAN | VNPRC_12103 | KIEN GIANG | IMP | IR | I | I1 |
| G171 | NEP THAI | VNPRC_12104 | KIEN GIANG | TRAD | IR | I | I1 |
| G172 | VND 95-20 | VNPRC_12105 | KIEN GIANG | IMP | IR | I | I1 |
| G173 | TAM THOM TRUNG QUOC | VNPRC_12107 | KIEN GIANG | TRAD | IR | I | Im |
| G177 | CHAM HOM | VNPRC_12563 | HOA BINH | TRAD | na | J | J1 |
| G178 | KHAU CHINH PHU | VNPRC_12573 | HOA BINH | TRAD | na | J | J1 |
| G179 | BLAO PU LAU | VNPRC_12581 | HOA BINH | TRAD | na | J | J1 |
| G18 | NEP QUYT HAI DUONG | VNPRC_407 | HAI DUONG | TRAD | na | I | I4 |
| G180 | CA DUNG HAT | VNPRC_12637 | na | TRAD | na | I | I2 |
| G181 | BLAU PLAN PIENG | VNPRC_12970 | SON LA | TRAD | UP | I | I6 |
| G182 | KHAU MO | VNPRC_13008 | SON LA | TRAD | UP | I | I6 |
| G183 | KHAU PE LANH | VNPRC_13076 | SON LA | TRAD | UP | I | Im |
| G186 | KHAU NO | VNPRC_13309 | SON LA | TRAD | UP | I | Im |
| G187 | KHAU DUONG PHUONG | VNPRC_13320 | SON LA | TRAD | UP | J | J1 |
| G189 | KHAU NAM RINH | VNPRC_13362 | DIEN BIEN | TRAD | UP | I | I3 |
| G19 | ON | VNPRC_509 | na | TRAD | na | I | Im |
| G190 | PLE PHMA CHUA | VNPRC_13363 | DIEN BIEN | TRAD | UP | I | Im |
| G191 | KHAU TAN | VNPRC_13422 | DIEN BIEN | TRAD | RL | J | J2 |
| G192 | KHAU BAO THAI | VNPRC_13423 | DIEN BIEN | TRAD | RL | I | Im |
| G193 | BLE PE XA | VNPRC_13424 | DIEN BIEN | TRAD | UP | J | J1 |
| G194 | BLE BLAU LIA | VNPRC_13425 | DIEN BIEN | TRAD | UP | J | J1 |
| G195 | BLE BDE | VNPRC_13426 | DIEN BIEN | TRAD | UP | J | J1 |
| G196 | BLE CO PON | VNPRC_13427 | DIEN BIEN | TRAD | UP | m | m |
| G198 | BLE BLAU DA | VNPRC_13429 | DIEN BIEN | TRAD | UP | J | na |
| G2 | TA CO LAO CAI | VNPRC_58 | LAO CAI | TRAD | na | I | Im |
| G20 | TE LE HOA BINH | VNPRC_553 | HOA BINH | TRAD | na | I | I4 |
| G200 | CHA FU NU | VNPRC_13431 | LAI CHAU | TRAD | UP | J | J1 |
| G201 | CHA XU PHU LU | VNPRC_13435 | LAI CHAU | TRAD | UP | I | Im |
| G202 | NONG TO | VNPRC_13442 | LAI CHAU | TRAD | UP | J | J1 |
| G203 | PLAU CA BANH | VNPRC_14212 | DIEN BIEN | TRAD | UP | J | J1 |
| G204 | PLE DO | VNPRC_14215 | DIEN BIEN | TRAD | UP | J | J1 |
| G205 | BLE BLAU CHO | VNPRC_14251 | SON LA | TRAD | UP | I | I3 |
| G206 | BLE BLAU DO | VNPRC_14252 | SON LA | TRAD | UP | J | J1 |
| G207 | KHAU LUA | VNPRC_14278 | SON LA | TRAD | RL | m | m |
| G208 | KHAU BOONG LAM | VNPRC_14279 | SON LA | TRAD | RL | I | Im |
| G209 | BLE CHO | VNPRC_14386 | LAI CHAU | TRAD | UP | I | I6 |
| G21 | GIE TRANG HOA BINH | VNPRC_614 | HOA BINH | TRAD | na | I | I4 |
| G210 | KHAU LECH | VNPRC_14408 | LAO CAI | TRAD | UP | J | J1 |
| G211 | PLAU NGOANG PLAC | VNPRC_14587 | LAO CAI | TRAD | UP | I | Im |
| G212 | PLAU BULAT | VNPRC_14589 | LAO CAI | TRAD | UP | J | J1 |
| G214 | BLE BLAU DO | VNPRC_14596 | LAO CAI | TRAD | RL | J | Jm |
| G216 | TOM BEO BUA | VNPRC_14607 | LAO CAI | TRAD | RL | J | J1 |
| G217 | BLE BLAU SOA | VNPRC_14615 | LAO CAI | TRAD | RL | J | J1 |
| G219 | KHAU LA LANH | VNPRC_14792 | SON LA | TRAD | RL | I | Im |
| G22 | TRUNG TRANG TUYEN QUANG | VNPRC_760 | na | TRAD | na | I | I4 |
| G220 | PLE LA | VNPRC_T5300 | na | TRAD | UP | J | J1 |
| G221 | KHAU MAC CO | VNPRC_T5455 | na | TRAD | UP | J | J1 |
| G222 | PLE MA MU | VNPRC_T6404 | na | TRAD | UP | J | J1 |
| G223 | BLE BLAU TAN | VNPRC_T6794 | na | TRAD | UP | J | J1 |
| G24 | TAM XOAN HAI HAU | VNPRC_1048 | NAM DINH | TRAD | na | m | m |
| G25 | NEP VANG ONG LAC SON HB | VNPRC_1058 | HOA BINH | TRAD | na | J | J2 |
| G26 | KHAU CAI NOI | VNPRC_1325 | TAY BAC | TRAD | na | J | J1 |
| G27 | DOAN KET | VNPRC_1421 | BAC THAI | TRAD | na | I | na |
| G29 | NEP CON | VNPRC_1427 | BAC THAI | TRAD | UP | I | na |
| G299 | BLAO SINH SAI | VNPRC_4806 | HOA BINH | TRAD | na | J | J1 |
| G3 | AN TU VO DO | VNPRC_85 | na | TRAD | na | I | I4 |
| G30 | TIEU CHET 5 | VNPRC_1625 | CAN THO | TRAD | na | I | I2 |
| G300 | NANG QUOT BIEN | VNPRC_5863 | BAC LIEU | TRAD | na | I | I2 |
| G31 | NANG CHI | VNPRC_1629 | CAN THO | TRAD | na | I | I2 |
| G32 | NANG DUM | VNPRC_1633 | CAN THO | TRAD | na | I | Im |
| G35 | SANAKI | VNPRC_1638 | CAN THO | na | na | I | I2 |
| G36 | NANG TAY | VNPRC_1643 | CAN THO | TRAD | na | I | Im |
| G37 | NEP CAM | VNPRC_1845 | HA GIANG | TRAD | RL | I | Im |
| G38 | NEP NUONG | VNPRC_1849 | HA GIANG | TRAD | UP | J | J1 |
| G39 | NEP CAM | VNPRC_1851 | HA GIANG | TRAD | RL | I | Im |
| G4 | NHONG DO HAI DUONG | VNPRC_135 | HAI DUONG | TRAD | na | I | I4 |
| G40 | NEP DO | VNPRC_2307 | KIEN GIANG | TRAD | RL | I | I2 |
| G41 | LUA DO | VNPRC_2310 | KIEN GIANG | TRAD | RL | I | I2 |
| G42 | LUA HON COI | VNPRC_2313 | KIEN GIANG | TRAD | RL | I | I2 |
| G43 | THANH TUA | VNPRC_2315 | KIEN GIANG | TRAD | RL | I | I2 |
| G44 | MOT BUI | VNPRC_2332 | KIEN GIANG | TRAD | RL | I | na |
| G45 | NEP CUC | VNPRC_2367 | NINH BINH | TRAD | RL | J | J4 |
| G46 | NEP BA LAO | VNPRC_2368 | NAM DINH | TRAD | MG | J | J4 |
| G47 | NEP ONG LAO | VNPRC_2369 | NAM DINH | TRAD | MG | J | J4 |
| G48 | LUA NGOI | VNPRC_2371 | NAM DINH | TRAD | MG | J | J4 |
| G49 | DT10 | VNPRC_2395 | na | IMP | na | I | I1 |
| G5 | NHONG TRANG HAI PHONG | VNPRC_149 | HAI PHONG | TRAD | na | I | I4 |
| G50 | LUA NEP BA THANG DANG 1 | VNPRC_3323 | QUANG NAM | TRAD | UP | J | J2 |
| G51 | BA TRANG HUONG | VNPRC_3332 | QUANG NAM | TRAD | UP | I | I5 |
| G52 | BA TRANG HUONG | VNPRC_3334 | QUANG NAM | TRAD | UP | I | I5 |
| G53 | LUA CAN DO | VNPRC_3351 | na | TRAD | na | I | I6 |
| G54 | LUA LOC DO | VNPRC_3360 | QUANG NAM | TRAD | RL | I | I6 |
| G56 | LUA MAN | VNPRC_3363 | QUANG NAM | TRAD | RL | I | I5 |
| G57 | NEP GHIM HUONG | VNPRC_3364 | QUANG NAM | TRAD | RL | I | Im |
| G58 | NEP HUONG LANG | VNPRC_3368 | QUANG NAM | TRAD | MG | I | I6 |
| G59 | NEP MAM | VNPRC_3371 | QUANG NAM | TRAD | na | I | I6 |
| G6 | SOM GIAI HUNG YEN | VNPRC_170 | na | TRAD | na | I | I4 |
| G60 | NEP CAU | VNPRC_3400 | QUANG BINH | TRAD | RL | J | na |
| G61 | NEP RAN | VNPRC_3402 | QUANG BINH | TRAD | na | J | J2 |
| G62 | QUANG TRANG | VNPRC_3426 | QUANG TRI | TRAD | IR | I | I5 |
| G63 | CHIEM DO | VNPRC_3429 | QUANG TRI | TRAD | na | I | I4 |
| G64 | VEN DO | VNPRC_3433 | QUANG TRI | TRAD | IR | I | I5 |
| G65 | NUOC MAN DANG 1 | VNPRC_3443 | QUANG TRI | TRAD | RL | I | I5 |
| G66 | LUA MUOI | VNPRC_3483 | QUANG NGAI | TRAD | RL | I | na |
| G67 | LUA TRI DO DANG 2 | VNPRC_3485 | BINH DINH | TRAD | RL | I | Im |
| G68 | NEP 3 THANG | VNPRC_3487 | BINH DINH | TRAD | IR | m | m |
| G69 | COC MOI DANG 1 | VNPRC_3488 | BINH DINH | TRAD | RL | I | I6 |
| G7 | TE TRANG HOA BINH | VNPRC_172 | HOA BINH | TRAD | na | I | I4 |
| G70 | COC MOI DANG 2 | VNPRC_3489 | BINH DINH | TRAD | RL | I | Im |
| G71 | LUA THOM | VNPRC_3493 | BINH DINH | TRAD | RL | I | na |
| G72 | LUA CANG DANG 1 | VNPRC_3494 | BINH DINH | TRAD | RL | I | Im |
| G73 | LUA CANG DANG 1 | VNPRC_3495 | BINH DINH | TRAD | RL | I | I6 |
| G74 | NEP QUA CO RAU DANG 2 | VNPRC_3497 | BINH DINH | TRAD | RL | I | I6 |
| G75 | NEP QUA | VNPRC_3498 | BINH DINH | TRAD | RL | I | na |
| G77 | CANG KIEN DANG 1 | VNPRC_3506 | BINH DINH | TRAD | RL | I | I6 |
| G78 | CANG KIEN DANG 2 | VNPRC_3507 | BINH DINH | TRAD | RL | I | I6 |
| G79 | LUA DA DANG 2 | VNPRC_3508 | BINH DINH | TRAD | RL | I | I6 |
| G8 | CHON TU 502 HOC VIEN | VNPRC_175 | na | TRAD | na | I | I4 |
| G80 | BA KTONG | VNPRC_3517 | QUANG NGAI | TRAD | UP | J | J3 |
| G81 | BA DO DANG 1 | VNPRC_3519 | QUANG NGAI | TRAD | UP | m | m |
| G83 | NEP VANG | VNPRC_3522 | QUANG NGAI | TRAD | UP | J | Jm |
| G84 | BA CHO KTE | VNPRC_3525 | BINH DINH | TRAD | na | J | J3 |
| G85 | CHANH CHUI | VNPRC_3550 | THANH HOA | TRAD | MG | J | J4 |
| G86 | TAN NGAN | VNPRC_3588 | YEN BAI | TRAD | na | J | J2 |
| G87 | KHAU PAN PUA | VNPRC_3886 | na | TRAD | na | J | J1 |
| G88 | BLE MA MUA | VNPRC_3895 | na | TRAD | na | J | J1 |
| G89 | KHAU BO KHA | VNPRC_3947 | na | TRAD | na | J | J1 |
| G9 | LOC TRANG SOM PLAY CAU | VNPRC_200 | na | TRAD | na | I | I4 |
| G90 | BLAO CLIA | VNPRC_4812 | HOA BINH | TRAD | UP | J | J1 |
| G91 | BLAO CO KEN | VNPRC_4815 | HOA BINH | TRAD | UP | J | J1 |
| G92 | BLAO CO CAM | VNPRC_4820 | HOA BINH | TRAD | UP | J | J1 |
| G93 | PO LE PO LAU XA | VNPRC_5034 | NGHE AN | TRAD | UP | I | I5 |
| G94 | LUA DO | VNPRC_5111 | THUA THIEN HUE | TRAD | UP | I | I5 |
| G95 | LUA CHAM | VNPRC_5127 | NAM DINH | TRAD | RL | I | I4 |
| G96 | CHIEM RONG | VNPRC_6191 | NAM DINH | TRAD | IR | I | Im |
| G97 | TAM THOM | VNPRC_6199 | NAM DINH | TRAD | IR | m | na |
| G98 | NGOI TIA | VNPRC_6203 | NAM DINH | TRAD | RL | J | J4 |
| G99 | LUA CHAM BIEN | VNPRC_6234 | NINH BINH | TRAD | RL | I | I4 |
| IR64 | IR64 | IRGC_66970 | na | IMP | IR | I | I |
| NPB | NIPPONBARE | IRGC_12731 | na | IMP | IR | J | J |

na = no data available; TRAD = traditional; IMP= improved; UP = upland; RL = rainfed lowland; MG = mangrove; IR = irrigated;I = indica; J = japonica; Sub-pop: sub-populations as defined from the results of Phung et al. (2014)
