# Supplementary material for: Genome-wide association mapping for root traits in a panel of rice accessions from Vietnam
Source: BMC Plant Biol. 2016 Mar 10;16:64. doi: 10.1186/s12870-016-0747-y (PMC4785749; doi:10.1186/s12870-016-0747-y)
Supplement: Additional file 2: Table S2. — Mean, standard deviation, range, and CV of the whole panel for all traits measured. (DOCX 31 kb) [file 12870_2016_747_MOESM2_ESM.docx]

Table S2: Mean, standard deviation, range, and CV of the whole panel for all traits measured

| Traits | N | Mean | sd | Min | Max | CV |
| --- | --- | --- | --- | --- | --- | --- |
| LLGHT (cm) | 194 | 94.7 | 12.6 | 63.9 | 125.0 | 13.3 |
| TIL | 194 | 7.45 | 3.82 | 1.61 | 20.8 | 51.3 |
| SDW (g) | 194 | 5.661 | 2.114 | 1.283 | 13.670 | 37.3 |
| DEPTH (cm) | 194 | 69.2 | 4.0 | 53.4 | 76.8 | 5.8 |
| MRL (cm) | 194 | 85.9 | 5.9 | 69.6 | 99.4 | 6.8 |
| NCR | 194 | 91.9 | 30.2 | 32.5 | 176.8 | 32.8 |
| NR_T | 194 | 14.5 | 4.4 | 5.5 | 34.9 | 30.0 |
| THK (mm) | 194 | 0.769 | 0.105 | 0.488 | 0.999 | 13.7 |
| DW0020 (g) | 194 | 0.879 | 0.277 | 0.313 | 1.785 | 31.5 |
| DW2040 (g) | 194 | 0.452 | 0.170 | 0.128 | 1.025 | 37.5 |
| DW4060 (g) | 194 | 0.208 | 0.102 | 0.034 | 0.549 | 48.9 |
| DWB60 (g) | 194 | 0.096 | 0.060 | -0.005 | 0.364 | 62.6 |
| DRW (g) | 194 | 0.303 | 0.146 | 0.031 | 0.780 | 48.1 |
| RDW (g) | 194 | 1.635 | 0.549 | 0.472 | 3.164 | 33.6 |
| PDW (g) | 194 | 7.291 | 2.572 | 1.936 | 16.810 | 35.3 |
| SRP (%) | 194 | 54.8 | 6.6 | 37.7 | 83.1 | 12.1 |
| DRP (%) | 194 | 17.9 | 4.5 | 4.5 | 29.3 | 25.3 |
| R_S | 194 | 0.3052 | 0.0626 | 0.1697 | 0.4968 | 20.5 |

N = final number of accessions; LLGTH = longest leaf length; TIL = number of tillers; SDW = shoot dry weight; DEPTH = deepest point reached by roots; MRL = maximum root length; NCR = number of crown roots; NR_T = number of crown root per tiller; THK = root thickness; DW0020 = root mass in the 00-20 cm segment; DW2040 = root mass in the 20-40 cm segment ; DW4060 = root mass in the 40-60 cm segment; DWB60 = root mass below 60 cm; DRW = deep root mass (<40 cm) weight; RDW = root dry weight; PDW = plant dry weight, SRP = shallow root proportion (0-20 cm); DRP = deep root proportion (<40 cm); R_S = root to shoot ratio.
