# Supplementary material for: Genome-wide association mapping for root traits in a panel of rice accessions from Vietnam
Source: BMC Plant Biol. 2016 Mar 10;16:64. doi: 10.1186/s12870-016-0747-y (PMC4785749; doi:10.1186/s12870-016-0747-y)
Supplement: Additional file 3: Figure S1. — Graphical representation of the panel. (PPTX 418 kb) [file 12870_2016_747_MOESM3_ESM.pptx]

## Slide 1
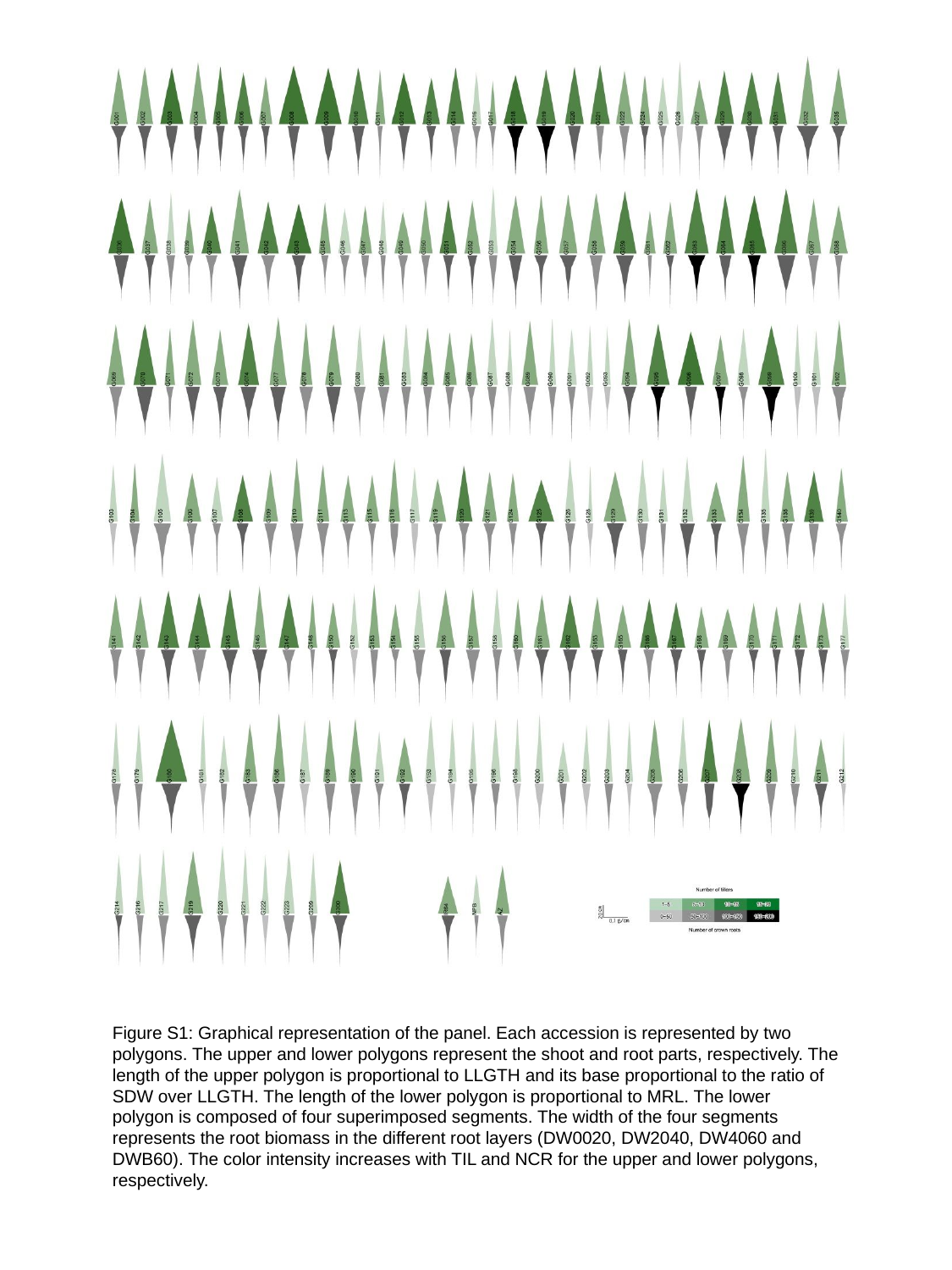

Figure S1: Graphical representation of the panel. Each accession is represented by two
polygons. The upper and lower polygons represent the shoot and root parts, respectively. The
length of the upper polygon is proportional to LLGTH and its base proportional to the ratio of
SDW over LLGTH. The length of the lower polygon is proportional to MRL. The lower
polygon is composed of four superimposed segments. The width of the four segments
represents the root biomass in the different root layers (DW0020, DW2040, DW4060 and
DWB60). The color intensity increases with TIL and NCR for the upper and lower polygons,
respectively.
