# Supplementary material for: Genome-wide association mapping for root traits in a panel of rice accessions from Vietnam
Source: BMC Plant Biol. 2016 Mar 10;16:64. doi: 10.1186/s12870-016-0747-y (PMC4785749; doi:10.1186/s12870-016-0747-y)
Supplement: Additional file 4: Table S3. — Mean comparisons between groups in the whole panel and subpopulations in each subpanel and percentage of variance explained by the structure (% var). (DOCX 48 kb) [file 12870_2016_747_MOESM4_ESM.docx]

Table S3: Mean comparisons between groups in the whole panel and subpopulations in each subpanel and percentage of variance explained by the structure (% var).

| Whole panel | LLGTH |  | TIL |  | SDW |  | DEPTH |  | MRL |  | NCR |  | NR_T |  | THK |  | DW0020 |  |
| --- | --- | --- | --- | --- | --- | --- | --- | --- | --- | --- | --- | --- | --- | --- | --- | --- | --- | --- |
| I | 93.4 | ab | 9.3 | a | 6.5906 | a | 69.0 | a | 86.2 | a | 106.2 | a | 13.5 | b | 0.746 | b | 0.9935 | a |
| J | 98.0 | a | 4.2 | c | 4.0581 | b | 69.4 | a | 85.3 | a | 64.6 | b | 16.1 | ab | 0.815 | a | 0.6799 | b |
| m | 85.3 | b | 7.2 | b | 5.0773 | b | 69.8 | a | 85.4 | a | 106.6 | a | 16.9 | a | 0.709 | b | 0.8266 | b |
| P | 0.0071 |  | <.0001 |  | <.0001 |  | 0.7291 |  | 0.6236 |  | <.0001 |  | 0.0001 |  | <.0001 |  | <.0001 |  |
| % var | 1.7 |  | 37.2 |  | 32.1 |  | 0.1 |  | 1.1 |  | 40.9 |  | 7.5 |  | 7.4 |  | 31.0 |  |
| Indica | LLGTH |  | TIL |  | SDW |  | DEPTH |  | MRL |  | NCR |  | NR_T |  | THK |  | DW0020 |  |
| I1 | 71.7 | d | 7.2 | bc | 4.7782 | b | 67.4 | a | 83.8 | b | 107.5 | ab | 17.4 | ab | 0.694 | bc | 0.8002 | a |
| I2 | 90.5 | c | 10.5 | b | 7.0594 | ab | 69.4 | a | 86.0 | b | 103.1 | ab | 11.4 | cd | 0.776 | ab | 1.0434 | a |
| I3 | 107.5 | a | 5.1 | c | 6.1245 | ab | 71.8 | a | 95.3 | a | 88.0 | c | 18.3 | a | 0.797 | a | 1.0402 | a |
| I4 | 95.8 | bc | 13.3 | a | 8.0163 | a | 67.5 | a | 85.4 | b | 120.4 | a | 10.2 | d | 0.675 | c | 0.9754 | a |
| I5 | 97.4 | abc | 9.5 | b | 6.3130 | ab | 68.0 | a | 86.5 | b | 119.5 | a | 14.5 | abc | 0.646 | c | 0.8889 | a |
| I6 | 103.8 | ab | 7.5 | bc | 6.5414 | ab | 70.7 | a | 90.2 | b | 92.9 | ab | 13.9 | bcd | 0.823 | a | 1.0256 | a |
| Im | 92.9 | bc | 7.9 | bc | 6.1568 | ab | 69.6 | a | 85.1 | b | 104.4 | ab | 15.1 | abc | 0.769 | ab | 1.0384 | a |
| P | <.0001 |  | <.0001 |  | 0.0036 |  | 0.0865 |  | 0.0008 |  | 0.0171 |  | <.0001 |  | <.0001 |  | 0.2232 |  |
| % var | 54.2 |  | 44.4 |  | 17.6 |  | 11.2 |  | 27.8 |  | 17.2 |  | 39.2 |  | 42.3 |  | 8.9 |  |
| Japonica | LLGTH |  | TIL |  | SDW |  | DEPTH |  | MRL |  | NCR |  | NR_T |  | THK |  | DW0020 |  |
| J1 | 103.4 | a | 3.7 | a | 3.8922 | a | 70.2 | a | 86.7 | ab | 56.1 | b | 16.1 | a | 0.855 | ab | 0.6827 | a |
| J2 | 82.3 | b | 5.2 | a | 4.1935 | a | 69.2 | a | 82.3 | bc | 86.3 | a | 17.2 | a | 0.701 | c | 0.6551 | a |
| J3 | 99.8 | a | 5.0 | a | 4.5120 | a | 72.9 | a | 90.5 | a | 65.5 | b | 13.8 | a | 0.921 | a | 0.7538 | a |
| J4 | 86.7 | b | 4.7 | a | 4.2690 | a | 63.5 | b | 77.2 | c | 75.0 | ab | 16.9 | a | 0.672 | c | 0.6426 | a |
| Jm | 101.4 | a | 4.8 | a | 4.5072 | a | 69.7 | a | 86.1 | ab | 67.7 | ab | 15.4 | a | 0.827 | b | 0.7521 | a |
| P | <.0001 |  | 0.0146 |  | 0.6128 |  | 0.0004 |  | 0.0002 |  | <.0001 |  | 0.6921 |  | <.0001 |  | 0.6918 |  |
| % var | 56.8 |  | 17.8 |  | 4.8 |  | 28.5 |  | 29.4 |  | 45.9 |  | 11.5 |  | 62.0 |  | 4.9 |  |

| Wholepanel | DW2040 |  | DW4060 |  | DWB60 |  | DRW |  | RDW |  | SRP |  | DRP |  | PDW |  | R_S |  |
| --- | --- | --- | --- | --- | --- | --- | --- | --- | --- | --- | --- | --- | --- | --- | --- | --- | --- | --- |
| I | 0.5030 | a | 0.2407 | a | 0.1018 | a | 0.3424 | a | 1.8390 | a | 55.3 | b | 17.9 | a | 8.4201 | a | 0.30 | a |
| J | 0.3690 | b | 0.1551 | b | 0.0877 | a | 0.2427 | b | 1.2916 | b | 53.4 | b | 18.2 | a | 5.3512 | b | 0.33 | a |
| m | 0.3831 | b | 0.1432 | b | 0.0702 | b | 0.1435 | b | 1.4231 | b | 59.7 | a | 14.8 | a | 6.4986 | b | 0.29 | a |
| P | <.0001 |  | <.0001 |  | 0.1576 |  | <.0001 |  | <.0001 |  | 0.0236 |  | 0.1589 |  | <.0001 |  | 0.0004 |  |
| % var | 15.7 |  | 17.2 |  | 2.3 |  | 12.1 |  | 24.7 |  | 1.5 |  | 0.1 |  | 32.5 |  | 6.1 |  |
| Indica | DW2040 |  | DW4060 |  | DWB60 |  | DRW |  | RDW |  | SRP |  | DRP |  | PDW |  | R_S |  |
| I1 | 0.3272 | c | 0.1493 | b | 0.0643 | c | 0.2136 | c | 1.3410 | c | 60.2 | a | 15.4 | c | 6.1200 | b | 0.29 | bcd |
| I2 | 0.4937 | ab | 0.2256 | ab | 0.0954 | bc | 0.3209 | bc | 1.8580 | abc | 56.9 | a | 16.9 | c | 8.9148 | ab | 0.28 | bcd |
| I3 | 0.5871 | a | 0.3365 | a | 0.2217 | a | 0.5582 | a | 2.1856 | a | 47.2 | c | 25.6 | a | 8.3120 | ab | 0.36 | a |
| I4 | 0.5149 | ab | 0.2711 | a | 0.0930 | bc | 0.3641 | bc | 1.8545 | abc | 53.3 | ab | 19.4 | bc | 9.8672 | a | 0.24 | d |
| I5 | 0.3890 | bc | 0.1621 | b | 0.0807 | bc | 0.2428 | c | 1.5206 | bc | 59.4 | a | 15.5 | c | 7.8425 | ab | 0.26 | cd |
| I6 | 0.6056 | a | 0.2901 | a | 0.1497 | b | 0.4397 | b | 2.0710 | ab | 49.5 | bc | 21.2 | b | 8.6138 | ab | 0.33 | ab |
| Im | 0.5165 | ab | 0.2359 | ab | 0.0985 | bc | 0.3344 | bc | 1.8893 | abc | 56.9 | a | 16.6 | c | 8.0166 | ab | 0.32 | abc |
| P | 0.0025 |  | 0.001 |  | <.0001 |  | <.0001 |  | 0.016 |  | <.0001 |  | <.0001 |  | 0.0118 |  | <.0001 |  |
| % var | 27.6 |  | 31.7 |  | 35.5 |  | 37.7 |  | 22.1 |  | 39.5 |  | 40.0 |  | 16.3 |  | 41.7 |  |
| Japonica | DW2040 |  | DW4060 |  | DWB60 |  | DRW |  | RDW |  | SRP |  | DRP |  | PDW |  | R_S |  |
| J1 | 0.3986 | a | 0.1625 | ab | 0.0972 | b | 0.2597 | b | 1.3410 | b | 51.2 | b | 19.2 | b | 5.2314 | a | 0.36 | a |
| J2 | 0.2533 | b | 0.0982 | b | 0.0541 | bc | 0.1523 | c | 1.0607 | b | 61.4 | a | 14.5 | c | 5.2600 | a | 0.26 | b |
| J3 | 0.4384 | a | 0.2289 | a | 0.1587 | a | 0.3876 | a | 1.5798 | a | 47.7 | b | 25.4 | a | 6.0840 | a | 0.35 | a |
| J4 | 0.3016 | ab | 0.0989 | b | 0.0196 | c | 0.1184 | c | 1.0626 | b | 59.9 | a | 11.5 | c | 5.3283 | a | 0.26 | b |
| Jm | 0.4409 | a | 0.2009 | a | 0.0977 | b | 0.2985 | b | 1.4914 | ab | 51.0 | b | 19.1 | b | 6.0067 | a | 0.35 | a |
| P | 0.0009 |  | <.0001 |  | <.0001 |  | <.0001 |  | 0.0119 |  | <.0001 |  | <.0001 |  | 0.6049 |  | <.0001 |  |
| % var | 33.7 |  | 38.4 |  | 46.7 |  | 50.9 |  | 24.2 |  | 56.1 |  | 49.0 |  | 5.5 |  | 48.5 |  |

P = probability of significance of the group effect (subpanels in whole panel; subpopulations in subpanel). % var = % of the phenotypic variation explained by the model obtained by regression of the phenotype on the percentage of admixture of each accession; I= indica; J = japonica; m= admixed. Within a column (separated for subpanel and each subpopulation), means followed by the same letters are not significantly different at P=0.05.
