# Supplementary material for: Genome-wide association mapping for root traits in a panel of rice accessions from Vietnam
Source: BMC Plant Biol. 2016 Mar 10;16:64. doi: 10.1186/s12870-016-0747-y (PMC4785749; doi:10.1186/s12870-016-0747-y)
Supplement: Additional file 5: Table S4. — Pearson correlation coefficients between traits in the whole panel (below the diagonal). Probabilities above the diagonal (in bold, significant at P < 0.05). W = whole panel; I = indica panel; J = japonica panel. (DOCX 43 kb) [file 12870_2016_747_MOESM5_ESM.docx]

Table S4: Pearson correlation coefficients between traits in the whole panel (below the diagonal). Probabilities above the diagonal (in bold, significant at P<0.05). W= whole panel; I = indica panel; J= japonica panel

| Variables |  | LLGHT | TIL | SDW | DEPTH | MRL | NCR | NR_T | THK | RDW | DRW | PDW | DRP | SRP | R_S |
| --- | --- | --- | --- | --- | --- | --- | --- | --- | --- | --- | --- | --- | --- | --- | --- |
| LLGHT | W | **1** | **0.010** | **0.002** | **< 0.0001** | **< 0.0001** | **0.013** | 0.160 | **< 0.0001** | **< 0.0001** | **< 0.0001** | **< 0.0001** | **< 0.0001** | **< 0.0001** | **< 0.0001** |
| LLGHT | I | **1** | 0.414 | **< 0.0001** | **0.001** | **< 0.0001** | 0.666 | 0.161 | **< 0.0001** | **< 0.0001** | **< 0.0001** | **< 0.0001** | **< 0.0001** | **< 0.0001** | **0.013** |
| LLGHT | J | **1** | **0.028** | **0.030** | **0.002** | **0.000** | **0.002** | 0.759 | **< 0.0001** | **< 0.0001** | **< 0.0001** | **0.004** | **< 0.0001** | **< 0.0001** | **0.001** |
| TIL | W | **-0.184** | **1** | **< 0.0001** | 0.456 | 0.683 | **< 0.0001** | **< 0.0001** | **< 0.0001** | **< 0.0001** | **< 0.0001** | **< 0.0001** | 0.789 | 0.429 | **< 0.0001** |
| TIL | I | -0.075 | **1** | **< 0.0001** | 0.714 | 0.196 | **< 0.0001** | **< 0.0001** | **0.033** | **0.000** | **0.046** | **< 0.0001** | 0.813 | 0.363 | **< 0.0001** |
| TIL | J | **-0.271** | **1** | **< 0.0001** | 0.763 | 0.575 | **< 0.0001** | **< 0.0001** | 0.209 | **0.000** | 0.076 | **< 0.0001** | 0.657 | 0.389 | **0.011** |
| SDW | W | **0.222** | **0.795** | **1** | **0.044** | **0.003** | **< 0.0001** | **< 0.0001** | 0.507 | **< 0.0001** | **< 0.0001** | **< 0.0001** | **0.003** | 0.079 | **< 0.0001** |
| SDW | I | **0.427** | **0.702** | **1** | **0.017** | **0.010** | **< 0.0001** | **< 0.0001** | 0.056 | **< 0.0001** | **< 0.0001** | **< 0.0001** | **0.000** | **0.001** | **< 0.0001** |
| SDW | J | **0.267** | **0.687** | **1** | **0.032** | 0.167 | **< 0.0001** | **0.043** | 0.317 | **< 0.0001** | **< 0.0001** | **< 0.0001** | 0.130 | 0.462 | **0.024** |
| DEPTH | W | **0.321** | -0.054 | **0.145** | **1** | **< 0.0001** | 0.650 | 0.979 | **< 0.0001** | **< 0.0001** | **< 0.0001** | **0.011** | **< 0.0001** | **0.000** | **0.014** |
| DEPTH | I | **0.310** | -0.034 | **0.216** | **1** | **< 0.0001** | 0.773 | 0.690 | **0.002** | **< 0.0001** | **< 0.0001** | **0.005** | **0.000** | **0.005** | 0.104 |
| DEPTH | J | **0.369** | 0.038 | **0.265** | **1** | **< 0.0001** | 0.853 | 0.352 | **0.000** | **< 0.0001** | **< 0.0001** | **0.007** | **< 0.0001** | **0.008** | 0.088 |
| MRL | W | **0.418** | -0.029 | **0.210** | **0.463** | **1** | 0.459 | 0.482 | **< 0.0001** | **< 0.0001** | **< 0.0001** | **0.000** | **< 0.0001** | **< 0.0001** | **0.002** |
| MRL | I | **0.459** | -0.118 | **0.232** | **0.390** | **1** | 0.497 | **0.047** | **0.002** | **< 0.0001** | **< 0.0001** | **0.002** | **< 0.0001** | **< 0.0001** | **0.004** |
| MRL | J | **0.438** | -0.070 | 0.172 | **0.609** | **1** | 0.169 | 0.420 | **< 0.0001** | **0.001** | **< 0.0001** | 0.056 | **< 0.0001** | **0.002** | **0.033** |
| NCR | W | **-0.178** | **0.715** | **0.714** | -0.033 | 0.053 | **1** | 0.342 | **< 0.0001** | **< 0.0001** | **< 0.0001** | **< 0.0001** | 0.114 | **< 0.0001** | **< 0.0001** |
| NCR | I | 0.040 | **0.535** | **0.612** | 0.026 | 0.062 | **1** | 0.616 | **0.018** | **< 0.0001** | **0.004** | **< 0.0001** | 0.759 | 0.130 | **0.001** |
| NCR | J | **-0.377** | **0.540** | **0.462** | -0.023 | -0.171 | **1** | **0.000** | **< 0.0001** | 0.421 | 0.384 | **0.001** | **0.020** | **< 0.0001** | **< 0.0001** |
| NR_T | W | 0.101 | **-0.660** | **-0.416** | 0.002 | 0.051 | -0.069 | **1** | 0.626 | **0.002** | **0.014** | **< 0.0001** | 0.161 | **0.009** | **< 0.0001** |
| NR_T | I | 0.128 | **-0.738** | **-0.363** | 0.037 | **0.181** | 0.046 | **1** | 0.242 | 0.654 | 0.996 | **0.001** | 0.804 | 0.108 | **< 0.0001** |
| NR_T | J | -0.038 | **-0.471** | **-0.250** | -0.116 | -0.101 | **0.425** | **1** | **0.017** | **0.019** | **0.010** | **0.028** | 0.052 | **0.001** | 0.361 |
| THK | W | **0.525** | **-0.318** | -0.048 | **0.329** | **0.303** | **-0.428** | 0.035 | **1** | **< 0.0001** | **< 0.0001** | 0.748 | **< 0.0001** | **< 0.0001** | **< 0.0001** |
| THK | I | **0.463** | **-0.194** | 0.174 | **0.273** | **0.283** | **-0.214** | 0.107 | **1** | **< 0.0001** | **< 0.0001** | **0.004** | **0.000** | **< 0.0001** | **< 0.0001** |
| THK | J | **0.559** | -0.157 | 0.125 | **0.443** | **0.496** | **-0.503** | **-0.293** | **1** | **< 0.0001** | **< 0.0001** | 0.072 | **< 0.0001** | **< 0.0001** | **< 0.0001** |
| RDW | W | **0.437** | **0.513** | **0.814** | **0.297** | **0.378** | **0.537** | **-0.224** | **0.301** | **1** | **< 0.0001** | **< 0.0001** | **< 0.0001** | **< 0.0001** | 0.072 |
| RDW | I | **0.604** | **0.323** | **0.760** | **0.356** | **0.415** | **0.454** | -0.041 | **0.545** | **1** | **< 0.0001** | **< 0.0001** | **< 0.0001** | **< 0.0001** | **0.002** |
| RDW | J | **0.550** | **0.424** | **0.774** | **0.462** | **0.384** | 0.101 | **-0.288** | **0.489** | **1** | **< 0.0001** | **< 0.0001** | **0.000** | **0.001** | **0.004** |
| DRW | W | **0.585** | **0.328** | **0.661** | **0.401** | **0.552** | **0.312** | **-0.176** | **0.398** | **0.881** | **1** | **< 0.0001** | **< 0.0001** | **< 0.0001** | **0.007** |
| DRW | I | **0.691** | **0.182** | **0.631** | **0.432** | **0.600** | **0.258** | 0.000 | **0.532** | **0.882** | **1** | **< 0.0001** | **< 0.0001** | **< 0.0001** | **0.002** |
| DRW | J | **0.646** | 0.220 | **0.571** | **0.561** | **0.550** | -0.109 | **-0.317** | **0.601** | **0.851** | **1** | **< 0.0001** | **< 0.0001** | **< 0.0001** | **0.003** |
| PDW | W | **0.277** | **0.761** | **0.992** | **0.183** | **0.254** | **0.697** | **-0.389** | 0.023 | **0.878** | **0.728** | **1** | **0.000** | **0.016** | **< 0.0001** |
| PDW | I | **0.488** | **0.650** | **0.990** | **0.257** | **0.284** | **0.599** | **-0.309** | **0.260** | **0.842** | **0.712** | **1** | **< 0.0001** | **< 0.0001** | **0.007** |
| PDW | J | **0.349** | **0.651** | **0.987** | **0.330** | 0.236 | **0.392** | **-0.271** | 0.223 | **0.865** | **0.667** | **1** | **0.036** | 0.160 | 0.286 |
| DRP | W | **0.591** | -0.019 | **0.215** | **0.398** | **0.631** | -0.114 | -0.101 | **0.422** | **0.383** | **0.740** | **0.257** | **1** | **< 0.0001** | **0.004** |
| DRP | I | **0.604** | 0.022 | **0.325** | **0.330** | **0.683** | -0.028 | -0.023 | **0.346** | **0.469** | **0.796** | **0.371** | **1** | **< 0.0001** | 0.066 |
| DRP | J | **0.556** | -0.056 | 0.188 | **0.536** | **0.607** | **-0.285** | -0.241 | **0.553** | **0.435** | **0.823** | **0.259** | **1** | **< 0.0001** | **0.026** |
| SRP | W | **-0.607** | 0.057 | -0.126 | **-0.271** | **-0.446** | **0.287** | **0.188** | **-0.510** | **-0.330** | **-0.625** | **-0.172** | **-0.840** | **1** | **< 0.0001** |
| SRP | I | **-0.594** | -0.083 | **-0.302** | **-0.256** | **-0.540** | 0.138 | 0.147 | **-0.417** | **-0.470** | **-0.717** | **-0.349** | **-0.866** | **1** | **0.004** |
| SRP | J | **-0.575** | 0.108 | -0.092 | **-0.326** | **-0.381** | **0.529** | **0.393** | **-0.615** | **-0.406** | **-0.705** | -0.175 | **-0.801** | **1** | **0.000** |
| R_S | W | **0.323** | **-0.517** | **-0.415** | **0.177** | **0.220** | **-0.441** | **0.301** | **0.575** | 0.129 | **0.192** | **-0.315** | **0.208** | **-0.338** | **1** |
| R_S | I | **0.226** | **-0.535** | **-0.363** | 0.149 | **0.259** | **-0.292** | **0.456** | **0.509** | **0.275** | **0.284** | **-0.243** | 0.168 | **-0.260** | **1** |
| R_S | J | **0.414** | **-0.310** | **-0.278** | 0.212 | **0.263** | **-0.528** | -0.114 | **0.558** | **0.346** | **0.362** | -0.133 | **0.274** | **-0.417** | **1** |

Traits DW0020, DW2040, DW4060, DWB60 not shown because of the table size.

LLGTH = longest leaf length; TIL = number of tillers; SDW = shoot dry weight; DEPTH = deepest point reached by roots; MRL = maximum root length; NCR = number of crown roots; NR_T = number of crown root per tiller; THK = root thickness; DW0020 = root mass in the 00-20 cm segment; DW2040 = root mass in the 20-40 cm segment ; DW4060 = root mass in the 40-60 cm segment; DWB60 = root mass below 60 cm; DRW = deep root mass (<40 cm) weight; RDW = root dry weight; PDW = plant dry weight, SRP = shallow root proportion (0-20 cm); DRP = deep root proportion (<40 cm); R_S = root to shoot ratio.
