# Supplementary material for: Genome-wide association mapping for root traits in a panel of rice accessions from Vietnam
Source: BMC Plant Biol. 2016 Mar 10;16:64. doi: 10.1186/s12870-016-0747-y (PMC4785749; doi:10.1186/s12870-016-0747-y)
Supplement: Additional file 6: Table S5. — List of genes with function located nearby the QTLs. (PDF 146 kb) [file 12870_2016_747_MOESM6_ESM.pdf]

Table S5: List of genes with function located nearby the QTLs

QTL	Chr.	Site 1	Site 2	Traits	Gene code	Annotation	Arabidopsis
	1	139 820	141 588	MRL, DWB6	**Os01g01290**	histone-like transcription factor and archaeal histone, putative, expressed	no
	**1**	145 577	147 852	MRL, DWB6	**Os01g01302**	shikimate kinase, putative, expressed	**AT3G26900.1**
**q33**	**1**	**146 251**		**MRL, DWB60,**			
	1	168 470	170 389	MRL, DWB6	**Os01g01340**	light-induced protein 1-like, putative, expressed	**AT3G26740.1**
	1	170 712	173 434	MRL, DWB6	**Os01g01350**	SNF7 domain containing protein, putative, expressed	no
	1	383 047	386 701	TIL	**Os01g01720**	pex14, putative, expressed	**AT5G62810.1**
	1	390 051	390 623	TIL	**Os01g01730**	serine/arginine repetitive matrix protein 2, putative, expressed	no
	1	392 081	396 651	TIL	**Os01g01740**	protein kinase domain containing protein, expressed	no
**q4**	**1**	**409 167**	**439 393**	**TIL**			
	1	409 700	410 818	TIL	**Os01g01770**	ZOS1-01 - C2H2 zinc finger protein, expressed	no
	1	412 579	415 830	TIL	**Os01g01780**	exostosin family protein, putative, expressed	**AT4G16745.2**
	1	2 154 326	2 155 994	DRP	**Os01g04750**	B3 DNA binding domain containing protein, expressed	no
**q83**	**1**	**2 173 691**		**DRP**			
	1	2 215 586	2 222 837	DEPTH	**Os01g04814**	vacuolar protein sorting-associating protein 4B, putative, expressed	**AT2G27600.1**
	1	2 226 000	2 229 526	DEPTH	**Os01g04840**	vacuolar protein sorting-associating protein 4B, putative, expressed	no
**q17**	**1**	**2 231 961**	**2 243 573**	**DEPTH**			
	1	2 245 732	2 250 576	DEPTH	**Os01g04860**	DUF647 domain containing protein, putative, expressed	**AT5G01510.1**
	1	2 250 820	2 255 073	DEPTH	**Os01g04870**	WD domain, G-beta repeat domain containing protein, expressed	**AT5G58760.1**
	1	2 265 477	2 271 051	DEPTH	**Os01g04900**	peptidase M50 family protein, putative, expressed	**AT5G05740.1**
	1	2 663 539	2 664 072	DWB60	**Os01g05580**	Exo70 exocyst complex subunit family, putative, expressed	no
	1	2 665 412	2 666 458	DWB60	**Os01g05585**	metallothionein, putative, expressed	no
	1	2 669 952	2 672 924	DWB60	**Os01g05600**	NBS-LRR disease resistance protein, putative, expressed	no
	1	2 674 400	2 675 373	DWB60	**Os01g05610**	Core histone H2A/H2B/H3/H4 domain containing protein, putative, expressed	no
**q72**	**1**	**2 678 840**		**DWB60**			
	1	2 682 019	2 684 988	DWB60	**Os01g05620**	NBS-LRR disease resistance protein, putative, expressed	no
	1	2 707 498	2 711 428	NR_T	**Os01g05670**	3-oxo-5-alpha-steroid 4-dehydrogenase, putative, expressed	**AT3G55360.1**
**q48**	**1**	**2 710 978**	**2 880 907**	**NR_T**			
	1	2 713 412	2 716 075	NR_T	**Os01g05680**	geranylgeranyl transferase type-1 subunit beta, putative, expressed	**AT2G39550.1**
	1	2 752 218	2 753 012	NR_T	**Os01g05760**	PAS2, putative, expressed	no
	1	2 765 773	2 768 644	NR_T	**Os01g05790**	SLT1 protein, putative, expressed	**AT2G37570.1**
	1	2 770 140	2 774 609	NR_T	**Os01g05800**	inner membrane protein, putative, expressed	**AT2G28800.1**
	1	2 776 973	2 781 775	NR_T	**Os01g05810**	gamma-glutamyltranspeptidase 1 precursor, putative, expressed	**AT4G29210.1**
	**1**	2 783 189	2 790 454	NR_T	**Os01g05820**	gamma-glutamyltranspeptidase 1 precursor, putative, expressed	no
	1	2 800 436	2 803 742	NR_T	**Os01g05840**	oxidoreductase, short chain dehydrogenase/reductase family domain containing	**AT2G37540.1**
	1	2 805 584	2 808 152	NR_T	**Os01g05860**	PPR repeat domain containing protein, putative, expressed	**AT3G15200.1**
	1	2 810 313	2 815 017	NR_T	**Os01g05870**	receptor kinase, putative, expressed	no
	1	2 815 175	2 816 401	NR_T	**Os01g05880**	OsFBX1 - F-box domain containing protein, expressed	no
	1	2 830 325	2 836 425	NR_T	**Os01g05940**	receptor kinase, putative, expressed	no
	1	2 839 934	2 843 514	NR_T	**Os01g05960**	receptor kinase, putative, expressed	no
	1	2 844 429	2 858 134	NR_T	**Os01g05970**	OsFBO1 - F-box and other domain containing protein, expressed	no
	1	2 851 954	2 855 225	NR_T	**Os01g05980**	receptor kinase, putative, expressed	no
	1	2 862 560	2 867 272	NR_T	**Os01g06000**	PPR repeat containing protein, expressed	**AT5G59900.1**
	1	2 872 110	2 872 988	NR_T	**Os01g06010**	Core histone H2A/H2B/H3/H4 domain containing protein, putative, expressed	no
	1	2 877 647	2 882 686	NR_T	**Os01g06020**	aspartyl-tRNA synthetase, putative, expressed	**AT4G33760.1**
	1	2 896 685	2 898 069	NR_T	**Os01g06060**	gibberellin receptor GID1L2, putative, expressed	no
	1	4 395 154	4 397 000	TIL	**Os01g08760**	choline/ethanolamine kinase, putative, expressed	no
	1	4 397 652	4 402 081	TIL	**Os01g08770**	WD domain, G-beta repeat domain containing protein, expressed	**AT3G10530.1**
	**1**	4 402 620	4 407 292	TIL	**Os01g08780**	endonuclease/exonuclease/phosphatase family domain containing protein,	no
**q5**	**1**	**4 404 405**		**TIL**			
	1	4 415 416	4 416 132	TIL	**Os01g08790**	histone-like transcription factor and archaeal histone, putative, expressed	**AT5G43250.1**
	1	4 417 283	4 419 160	TIL	**Os01g08800**	cytochrome P450, putative, expressed	no
	1	4 421 318	4 423 107	TIL	**Os01g08810**	cytochrome P450, putative, expressed	no
	1	5 809 557	5 811 617	DWB60, DR	**Os01g10890**	CAMK_KIN1/SNF1/Nim1_like.8 - CAMK includes calcium/calmodulin depedent	**AT5G45820.1**
	1	5 814 778	5 819 207	DWB60, DR	**Os01g10900**	MRH1, putative, expressed	**AT4G18640.1**
	1	5 823 869	5 828 251	DWB60, DR	**Os01g10920**	peptide-N4-asparagine amidase A, putative, expressed	no
	1	5 828 553	5 830 832	DWB60, DR	**Os01g10930**	peptide-N4-asparagine amidase A, putative, expressed	**AT3G14920.1**
	1	5 833 613	5 834 107	DWB60, DR	**Os01g10940**	S-adenosylmethionine synthetase 2, putative, expressed	no
	1	5 837 475	5 839 898	DWB60, DR	**Os01g10950**	peptide-N4-asparagine amidase A, putative, expressed	no
	1	5 841 736	5 843 574	DWB60, DR	**Os01g10960**	peptide-N4-asparagine amidase A, putative, expressed	no
	1	5 843 574	5 848 945	DWB60, DR	**Os01g10970**	auxin-induced protein 5NG4, putative, expressed	no
**q73**	**1**	**5 851 606**		**DWB60, DRW**			
	1	5 856 467	5 857 920	DWB60, DR	**Os01g10980**	auxin-induced protein 5NG4, putative, expressed	no
	1	5 861 816	5 863 794	DWB60, DR	**Os01g10990**	integral membrane protein DUF6 containing protein, expressed	no
	1	5 875 786	5 877 849	DWB60, DR	**Os01g11010**	peptide-N4-asparagine amidase A, putative, expressed	no
	1	6 377 261	6 382 037	DRP	**Os01g11790**	GDSL-like lipase/acylhydrolase, putative, expressed	no
	1	6 387 691	6 391 467	DRP	**Os01g11810**	powdery mildew res	no
	1	6 396 150	6 397 294	DRP	**Os01g11830**	oryzain alpha chain precursor, putative, expressed	no
	1	6 400 890	6 402 066	DRP	**Os01g11840**	xylem cysteine proteinase 2 precursor, putative, expressed	no
**q84**	**1**	**6 404 307**		**DRP**			
	1	6 412 786	6 419 895	DRP	**Os01g11860**	DJ-1 family protein, putative, expressed	no
	1	10 291 889	10 295 536	NR_T	**Os01g18320**	protoporphyrinogen oxidase, chloroplast precursor, putative, expressed	**AT4G01690.1**
**q49**	**1**	**10 299 033**		**NR_T**			
	1	10 303 744	10 304 211	NR_T	**Os01g18340**	glycine-rich cell wall protein, putative, expressed	no
	1	10 312 341	10 314 826	NR_T	**Os01g18360**	OsIAA4 - Auxin-responsive Aux/IAA gene family member, expressed	no
**q55**	**1**	**15 990 976**	**17 419 950**	**THK**		**Too large fragment: no gene search**	
	1	17 697 080	17 706 376	DEPTH	**Os01g32280**	transmembrane protein, putative, expressed	**AT5G19930.1**
**q18**	**1**	**17 715 289**		**DEPTH**			
	1	19 264 464	19 280 458	THK	**Os01g34890**	GLUCAN SYNTHASE-LIKE protein, putative, expressed	no
	1	19 281 427	19 282 266	THK	**Os01g34900**	GLUCAN SYNTHASE-LIKE protein, putative, expressed	no
**q56**	**1**	**19 286 868**		**THK**			
	1	19 295 569	19 302 138	THK	**Os01g34920**	beta-galactosidase precursor, putative, expressed	**AT1G31740.1**

QTL	Chr.	Site 1	Site 2	Traits	Gene code	Annotation	Arabidopsis
	1	19 303 350	19 306 036	THK	**Os01g34930**	callose synthase, putative, expressed	no
	1	27 301 427	27 303 025	TIL	**Os01g47730**	ras-related protein, putative, expressed	no
	1	27 303 111	27 312 308	TIL	**Os01g47740**	zinc finger, C3HC4 type domain containing protein, expressed	no
	1	27 317 556	27 321 710	TIL	**Os01g47750**	splicing factor U2AF 35 kDa subunit, putative, expressed	no
**q6**	**1**	**27 325 298**		**TIL**			
	1	27 326 050	27 327 030	TIL	**Os01g47760**	OsGrx_I1 - glutaredoxin subgroup III, expressed	**AT1G28480.1**
	1	27 336 260	27 339 363	TIL	**Os01g47770**	CS domain containing protein, putative, expressed	**AT5G58740.1**
	1	28 555 935	28 560 699	NCR	**Os01g49690**	Ser/Thr protein phosphatase family protein, putative, expressed	**AT1G50370.1**
	1	28 566 625	28 567 663	NCR	**Os01g49710**	glutathione S-transferase, putative, expressed	no
	1	28 568 721	28 569 792	NCR	**Os01g49720**	glutathione S-transferase, putative, expressed	no
**q36**	**1**	**28 579 082**		**NCR**			
	1	28 582 843	28 586 527	NCR	**Os01g49770**	zinc finger, C3HC4 type domain containing protein, expressed	**AT1G17970.1**
	1	35 302 492	35 305 739	NCR	**Os01g61010**	nodulin, putative, expressed	no
**q37**	**1**	**35 307 113**	**35 377 267**	**NCR**			
	1	35 309 604	35 312 765	NCR	**Os01g61020**	curculin-like lectin family protein, putative, expressed	**AT3G51710.1**
	1	35 314 359	35 318 845	NCR	**Os01g61030**	VHS and GAT domain containing protein, expressed	no
	1	35 320 780	35 327 164	NCR	**Os01g61044**	transmembrane amino acid transporter protein, putative, expressed	**AT3G56200.1**
	1	35 328 776	35 331 222	NCR	**Os01g61060**	SAG20, putative, expressed	no
	1	39 085 081	39 091 029	DEPTH	**Os01g67330**	nucleotide-sugar transporter family protein, putative, expressed	no
	1	39 091 728	39 095 339	DEPTH	**Os01g67340**	STRUBBELIG-RECEPTOR FAMILY 8 precursor, putative, expressed	no
	1	39 099 009	39 101 767	DEPTH	**Os01g67350**	cysteine protease, putative, expressed	no
**q19**	**1**	**39 102 194**	**39 143 941**	**DEPTH**			
	1	39 102 276	39 105 764	DEPTH	**Os01g67360**	methyltransferase, putative, expressed	no
	1	39 136 937	39 139 380	DEPTH	**Os01g67390**	POEI32 - Pollen Ole e I allergen and extensin family protein precursor, expressed	no
	1	39 141 130	39 145 479	DEPTH	**Os01g67410**	AP2/EREBP transcription factor BABY BOOM, putative, expressed	no
	1	39 158 153	39 165 729	DEPTH	**Os01g67420**	lipase, putative, expressed	no
	1	42 689 893	42 696 058	NR_T	**Os01g73680**	aspartyl aminopeptidase, putative, expressed	**AT5G04710.1**
	1	42 695 091	42 699 059	NR_T	**Os01g73690**	pentatricopeptide, putative, expressed	**AT2G16650.1**
	1	42 699 971	42 701 323	NR_T	**Os01g73700**	RCLEA2 - Root cap and Late embryogenes	no
	1	42 704 282	42 705 375	NR_T	**Os01g73710**	RCLEA3 - Root cap and Late embryogenes	no
**q50**	**1**	**42 706 214**		**NR_T**			
	1	42 706 524	42 707 981	NR_T	**Os01g73720**	RCLEA4 - Root cap and Late embryogenes	no
	1	42 710 696	42 712 138	NR_T	**Os01g73730**	RCLEA5 - Root cap and Late embryogenes	no
	1	42 713 303	42 713 811	NR_T	**Os01g73740**	lipase, putative, expressed	no
	1	42 715 724	42 721 562	NR_T	**Os01g73750**	Leucine Rich Repeat family protein, expressed	**AT2G36370.1**
	1	42 723 514	42 726 767	NR_T	**Os01g73760**	IPP transferase, putative, expressed	**AT2G27760.1**
	1	42 727 426	42 728 363	NR_T	**Os01g73770**	dehydration-responsive element-binding protein, putative, expressed	no
	2	4 988 483	4 998 043	DW2040	**Os02g09720**	multidrug res	no
**q66**	**2**	**5 015 093**		**DW2040**			
	2	5 019 765	5 028 028	DW2040	**Os02g09740**	STRUBBELIG-RECEPTOR FAMILY 7 precursor, putative, expressed	**AT4G22130.1**
	2	5 028 562	5 030 420	DW2040	**Os02g09750**	lycopene beta cyclase, chloroplast precursor, putative, expressed	**AT3G10230.1**
	2	5 038 459	5 043 329	DW2040	**Os02g09770**	abhydrolase domain-containing protein FAM108C1, putative, expressed	no
	2	6 188 722	6 193 632	DEPTH	**Os02g11940**	sterol glucosyltransferase, putative, expressed	**AT5G24750.1**
	2	6 197 353	6 204 374	DEPTH	**Os02g11960**	ABC transporter, ATP-binding protein, putative, expressed	no
	2	6 208 684	6 212 210	DEPTH	**Os02g11980**	receptor-like protein kinase precursor, putative, expressed	no
**q20**	**2**	**6 217 128**		**DEPTH**			
	2	6 228 456	6 231 985	DEPTH	**Os02g12010**	receptor-like protein kinase precursor, putative, expressed	no
	2	10 490 446	10 496 413	TIL	**Os02g18070**	NB-ARC domain containing protein, expressed	no
	**2**	10 503 049	10 508 541	TIL	**Os02g18080**	NB-ARC domain containing protein, expressed	no
**q7**	**2**	**10 505 253**		**TIL**			
	2	14 355 563	14 356 170	DRW	**Os02g24740**	OsSAUR9 - Auxin-responsive SAUR gene family member, expressed	no
**q78**	**2**	**14 366 103**		**DRW**			
	2	23 527 518	23 530 251	DWB60	**Os02g38920**	glyceraldehyde-3-phosphate dehydrogenase, putative, expressed	no
	2	23 531 392	23 533 277	DWB60	**Os02g38930**	cytochrome P450 71D7, putative, expressed	no
	2	23 535 117	23 536 974	DWB60	**Os02g38940**	cytochrome P450, putative, expressed	no
	2	23 538 347	23 540 253	DWB60	**Os02g38950**	PPR repeat containing protein, expressed	**AT3G04130.1**
	**2**	**23 549 174**	**23 554 382**	**DWB60**	**Os02g38980**	bromodomain domain containing protein, expressed	**AT5G14270.2**
**q74**	**2**	**23 550 168**		**DWB60**			
	2	23 559 740	23 561 669	DWB60	**Os02g39000**	remorin C-terminal domain containing protein, putative, expressed	no
	2	23 562 276	23 566 153	DWB60	**Os02g39010**	cyclin-dependent kinase G-1, putative, expressed	no
	2	23 572 961	23 575 571	DWB60	**Os02g39030**	transmembrane protein 17, putative, expressed	no
	2	25 967 752	25 968 734	DW0020	**Os02g43120**	zinc finger, C3HC4 type domain containing protein, expressed	no
	2	25 972 448	25 978 887	DW0020	**Os02g43130**	kinesin motor domain containing protein, expressed	no
**q64**	**2**	**25 990 556**		**DW0020**			
	2	25 996 914	25 999 176	DW0020	**Os02g43150**	GATA zinc finger domain containing protein, expressed	no
	2	30 736 687	30 743 642	DWB60	**Os02g50340**	membrane attack complex component/perforin/complement C9, putative,	**AT1G14780.1**
	2	30 745 336	30 749 371	DWB60	**Os02g50350**	dihydroorotate dihydrogenase protein, putative, expressed	**AT3G17810.1**
	2	30 750 564	30 756 536	DWB60	**Os02g50360**	nicalin, putative, expressed	**AT3G52640.2**
	2	30 756 675	30 762 768	DWB60	**Os02g50370**	helicase domain-containing protein, putative, expressed	**AT1G33390.1**
**q75**	**2**	**30 762 847**		**DWB60**			
	2	31 636 492	31 638 258	NCR	**Os02g51660**	trpH, putative, expressed	no
	2	31 648 468	31 650 538	NCR	**Os02g51670**	ethylene-responsive transcription factor, putative, expressed	**AT1G78080.1**
**q38**	**2**	**31 652 149**		**NCR**			
	2	31 663 080	31 665 818	NCR	**Os02g51680**	uncharacterized glycosyl hydrolase Rv2006/MT2062, putative, expressed	no
	2	31 676 446	31 677 556	NCR	**Os02g51710**	wound/stress protein, putative, expressed	**AT4G39730.1**
	2	35 427 552	35 430 640	THK	**Os02g57854**	vacuolar ATP synthase subunit F, putative, expressed	**AT4G02620.1**
	2	35 431 032	35 432 840	THK	**Os02g57860**	OsFBX71 - F-box domain containing protein, expressed	no
	2	35 440 926	35 442 608	THK	**Os02g57890**	OsFBX72 - F-box domain containing protein, expressed	no
	2	35 447 595	35 449 965	THK	**Os02g57910**	OsFBX73 - F-box domain containing protein, expressed	no
**q57**	**2**	**35 453 974**		**THK**			
	2	35 498 222	35 501 036	THK	**Os02g57960**	Leucine Rich Repeat family protein, expressed	no
	2	35 501 040	35 501 778	THK	**Os02g57965**	F-box domain containing protein, expressed	no
	2	35 502 083	35 507 050	THK	**Os02g57980**	DEAD/DEAH box helicase, putative, expressed	**AT2G07750.1**

QTL	Chr.	Site 1	Site 2	Traits	Gene code	Annotation	Arabidopsis
	2	35 507 155	35 510 880	THK	Os02g57990	methionine adenosyltransferase regulatory beta subunit-related, putative,	AT4G00560.4
q58	2	35 509 414	35 510 032	THK			
	2	35 517 531	35 523 358	THK	Os02g58020	ABC transporter, ATP-binding protein, putative, expressed	AT1G64550.1
	2	35 526 587	35 533 091	THK	Os02g58030	leucine-rich repeat-containing protein 40, putative, expressed	AT3G15410.2
	2	35 534 139	35 536 130	THK	Os02g58040	OsFBX75 - F-box domain containing protein, expressed	no
	3	3 525 078	3 531 357	LLGHT	Os03g06940	beta-galactosidase, putative, expressed	AT4G36360.1
	3	3 540 151	3 550 714	LLGHT	Os03g06950	ubiquitin carboxyl-terminal hydrolase domain containing protein, expressed	AT3G47890.1
	3	3 551 775	3 554 415	LLGHT	Os03g06960	vesicle-associated membrane protein, putative, expressed	no
	3	3 555 490	3 558 352	LLGHT	Os03g06970	tetratricopeptide repeat domain containing protein, expressed	no
q1	3	3 555 683		LLGHT			
	3	3 561 501	3 565 365	LLGHT	Os03g06980	nucleic acid binding protein, putative, expressed	AT3G07030.1
	3	3 566 777	3 568 821	LLGHT	Os03g06990	oxidoreductase, putative, expressed	no
	3	3 569 977	3 571 884	LLGHT	Os03g07000	oxidoreductase, putative, expressed	no
	3	14 513 696	14 518 548	NCR	Os03g25390	novel plant SNARE 11, putative, expressed	AT3G17440.1
	3	14 526 166	14 530 851	NCR	Os03g25400	kinase, putative, expressed	no
	3	14 538 130	14 539 609	NCR	Os03g25430	transcription regulator, putative, expressed	no
q39	3	14 543 326		NCR			
	3	14 545 715	14 547 929	NCR	Os03g25450	h/ACA ribonucleoprotein complex subunit 4, putative, expressed	no
	3	14 548 905	14 549 608	NCR	Os03g25460	OsRCI2-4 - Putative low temperature and salt responsive protein, expressed	no
	3	14 552 198	14 552 749	NCR	Os03g25470	ctr copper transporter family protein, putative, expressed	no
	3	14 553 102	14 556 229	NCR	Os03g25480	cytochrome P450, putative, expressed	no
	3	14 559 322	14 561 845	NCR	Os03g25490	cytochrome P450 72A1, putative, expressed	no
	3	14 567 492	14 571 763	NCR	Os03g25500	cytochrome P450 72A1, putative, expressed	no
	3	27 644 551	27 647 533	TIL, NCR	Os03g48480	thioesterase family protein, putative, expressed	AT1G48320.1
	3	27 652 240	27 659 735	TIL, NCR	Os03g48490	centromere protein, putative, expressed	AT4G38070.1
q8	3	27 657 195		TIL, NCR			
	3	27 662 425	27 663 177	TIL, NCR	Os03g48520	PB1 domain containing protein, expressed	no
	3	27 678 462	27 681 754	TIL, NCR	Os03g48560	N-acetylglucosaminyltransferase, putative, expressed	no
	3	29 766 132	29 774 184	TIL	Os03g51900	DEAD-box ATP-dependent RNA helicase, putative, expressed	AT4G34910.1
	3	29 774 810	29 776 910	TIL	Os03g51910	basic helix-loop-helix protein, putative, expressed	AT1G10120.1
	3	29 784 613	29 787 015	TIL	Os03g51920	peptidase, M50 family, putative, expressed	AT1G17870.1
q9	3	29 793 313		TIL			
	3	36 124 403	36 132 012	THK	Os03g63940	CBS domain containing membrane protein, putative, expressed	no
	3	36 132 038	36 134 636	THK	Os03g63950	plastid-specific 30S ribosomal protein 1, chloroplast precursor, putative,	AT5G24490.1
	3	36 150 664	36 152 517	THK	Os03g63970	gibberellin 20 oxidase 1, putative, expressed	AT5G51810.1
q59	3	36 156 421		THK			
q85	4	4 170 196		DRP			
	4	5 005 077	5 006 210	TIL	Os04g09390	HEV3 - Hevein family protein precursor, expressed	no
q10	4	5 029 726		TIL			
	4	5 040 350	5 043 003	TIL	Os04g09430	cytochrome P450, putative, expressed	no
q80	4	13 232 104		SRP			
	4	20 495 975	20 497 051	DEPTH	Os04g33830	membrane protein, putative, expressed	AT1G08380.1
	4	20 499 421	20 501 058	DEPTH	Os04g33840	pentatricopeptide, putative, expressed	AT5G37570.1
q21	4	20 517 263		DEPTH			
	4	20 519 152	20 520 509	DEPTH	Os04g33870	DUF623 domain containing protein, expressed	no
	4	20 526 065	20 528 257	DEPTH	Os04g33880	HVA22, putative, expressed	no
	4	20 534 071	20 534 774	DEPTH	Os04g33900	ctr copper transporter family protein, putative, expressed	no
	4	30 284 968	30 286 216	TIL	Os04g51140	E2F-related protein, putative, expressed	no
	4	30 297 864	30 305 606	TIL	Os04g51172	Disease resistance/zinc finger/chromosome condensation-like region protein,	no
q11	4	30 302 841		TIL			
	4	30 311 519	30 316 303	TIL	Os04g51180	GPR89A, putative, expressed	AT1G64990.1
	4	30 317 693	30 321 649	TIL	Os04g51190	growth-regulating factor, putative, expressed	no
	4	30 413 784	30 415 688	DW0020	Os04g51360	oxidoreductase, 2OG-Fe oxygenase family protein, putative, expressed	AT1G31600.1
	4	30 416 446	30 424 148	DW0020	Os04g51370	protein kinase, putative, expressed	no
	4	30 428 188	30 431 745	DW0020	Os04g51380	protein-S-	AT5G08335.1
	4	30 433 199	30 438 364	DW0020	Os04g51390	aldose 1-epimerase, putative, expressed	no
q65	4	30 438 406		DW0020			
	4	30 439 467	30 442 859	DW0020	Os04g51400	zinc finger, C3HC4 type domain containing protein, expressed	no
	5	7 398 476	7 403 351	NCR	Os05g13330	per1-like family protein, putative, expressed	AT1G16560.1
	5	7 418 020	7 422 194	NCR	Os05g13370	OsRhmbd12 - Putative Rhomboid homologue, expressed	AT5G25752.1
q40	5	7 422 947		NCR			
	5	7 446 336	7 450 295	NCR	Os05g13420	large tegument protein, putative, expressed	no
	5	16 184 117	16 186 813	NR_T	Os05g27790	membrane associated DUF588 domain containing protein, putative, expressed	AT2G38480.1
q51	5	16 205 923	16 458 100	NR_T			
	5	16 216 524	16 220 140	NR_T	Os05g27820	patellin protein, putative, expressed	AT3G51670.1
	5	16 267 416	16 273 970	NR_T	Os05g27880	BBF1 - 2 Bric-a-Brac, Tramtrack, Broad Complex BTB domains with a F5/8 type C d	AT2G30600.5
	5	16 289 244	16 296 693	NR_T	Os05g27920	phox domain-containing protein, putative, expressed	AT5G58440.1
	5	16 296 914	16 300 385	NR_T	Os05g27930	AP2 domain containing protein, expressed	no
	5	16 302 689	16 306 135	NR_T	Os05g27940	40S ribosomal protein S7, putative, expressed	no
	5	16 306 507	16 310 548	NR_T	Os05g27950	DUF538 domain containing protein, putative, expressed	AT3G08890.1
	5	16 320 971	16 321 771	NR_T	Os05g27970	POEI26 - Pollen Ole e I allergen and extensin family protein precursor, putative,	no
	5	16 327 595	16 328 435	NR_T	Os05g27980	DUF260 domain containing protein, putative, expressed	AT2G30130.1
	5	16 343 854	16 346 618	NR_T	Os05g28010	TATA-binding protein-associated factor TAFII55 family protein, putative, expressed	AT1G55300.2
	5	16 362 685	16 364 011	NR_T	Os05g28040	DUF640 domain containing protein, putative, expressed	no
	5	16 466 130	16 466 923	NR_T	Os05g28150	lipase, putative, expressed	no
	5	16 477 623	16 485 544	NR_T	Os05g28180	AMP deaminase, putative, expressed	no
	5	18 106 224	18 110 987	MRL	Os05g31140	glycosyl hydrolases family 17, putative, expressed	no
q34	5	18 109 976		MRL			
	5	18 126 333	18 129 661	MRL	Os05g31160	peptide chain release factor 2, putative, expressed	AT3G57190.1
	6	1 880 536	1 891 125	SRP	Os06g04399	hypersensitive-induced reaction protein 4, putative, expressed	no
	6	1 893 929	1 894 306	SRP	Os06g04420	lipoxygenase 4, putative	no
	6	1 900 862	1 909 008	SRP	Os06g04450	Sec1 family transport protein, putative, expressed	no
q81	6	1 901 540	1 930 717	SRP			

QTL	Chr.	Site 1	Site 2	Traits	Gene code	Annotation	Arabidopsis
	6	1 909 663	1 913 030	SRP	**Os06g04460**	hypersensitive-induced response protein, putative, expressed	**AT5G51570.1**
	6	1 927 256	1 930 883	SRP	**Os06g04500**	cornichon protein, putative, expressed	**AT1G12390.1**
	6	1 931 296	1 936 380	SRP	**Os06g04510**	enolase, putative, expressed	no
	6	1 949 424	1 953 325	SRP	**Os06g04530**	OsClp9 - Putative Clp protease homologue, expressed	**AT1G12410.1**
	6	3 994 993	3 996 159	DW2040	**Os06g08250**	zinc finger family protein, putative, expressed	no
	6	4 008 139	4 013 539	DW2040	**Os06g08280**	protein kinase domain containing protein, expressed	**AT3G46920.1**
	6	4 015 962	4 019 327	DW2040	**Os06g08290**	MYB family transcription factor, putative, expressed	no
	6	4 019 847	4 022 602	DW2040	Os06g08300	FAD dependent oxidoreductase domain containing protein, expressed	**AT2G22650.1**
**q67**	**6**	**4 021 017**		**DW2040**			
	6	4 027 361	4 030 375	DW2040	**Os06g08310**	plasma membrane ATPase, putative, expressed	no
	6	4 033 795	4 034 043	DW2040	**Os06g08320**	60S ribosomal protein L39, putative, expressed	no
	6	4 040 908	4 041 551	DW2040	**Os06g08340**	AP2 domain containing protein, expressed	no
	6	4 093 231	4 100 936	DW4060, R	**Os06g08400**	LIM domain-containing protein, putative, expressed	**AT4G36860.1**
**q69**	**6**	**4 127 010**		**DW4060, RDW, DRW**			
	6	4 137 248	4 142 663	DW4060, R	**Os06g08440**	two-component response regulator, putative, expressed	**AT2G25180.1**
	6	4 142 851	4 150 976	DW4060, R	**Os06g08450**	histidine kinase, putative, expressed	**AT2G47430.1**
	6	4 587 789	4 591 087	R_S	**Os06g09120**	glutamate receptor 2.8 precursor, putative, expressed	no
	6	4 593 689	4 597 978	R_S	**Os06g09130**	glutamate receptor 2.8 precursor, putative, expressed	no
**q86**	**6**	**4 601 154**		**R_S**			
	6	4 612 249	4 615 892	R_S	**Os06g09170**	zinc ion binding protein, putative, expressed	**AT1G55915.1**
	6	4 616 608	4 618 211	R_S	**Os06g09180**	OsMKK5 - putative MAPKK based on amino acid sequence homology, expressed	**AT1G51660.1**
	6	4 630 848	4 632 543	THK	**Os06g09210**	cytochrome P450 72A1, putative, expressed	no
	6	4 633 663	4 635 469	THK	**Os06g09220**	cytochrome P450 72A1, putative, expressed	no
	6	4 640 591	4 645 101	THK	**Os06g09230**	serine threonine kinase, putative, expressed	no
	**6**	**4 647 954**	**4 649 523**	**THK**	**Os06g09240**	anthocyanidin 3-O-glucosyltransferase, putative, expressed	**AT5G17050.1**
**q60**	**6**	**4 649 357**		**THK**			
	6	4 653 457	4 655 652	THK	**Os06g09270**	hypro1, putative, expressed	no
	6	4 659 377	4 667 061	THK	**Os06g09280**	ATP-dependent RNA helicase, putative, expressed	**AT1G26370.1**
	6	4 667 486	4 670 980	THK	**Os06g09290**	26S protease regulatory subunit 7, putative, expressed	**AT1G53750.1**
	6	7 825 468	7 829 696	LLGHT	**Os06g14040**	ThiF family domain containing protein, putative, expressed	**AT5G37530.1**
	**6**	7 835 233	7 841 307	**LLGHT**	**Os06g14050**	membrane attack complex component/perforin/complement C9, putative,	no
**q2**	**6**	**7 841 256**	**8 083 414**	**LLGHT**			
	6	7 841 699	7 844 834	LLGHT	**Os06g14060**	MBTB10 - Bric-a-Brac, Tramtrack, Broad Complex BTB domain with Meprin and	no
	6	7 845 545	7 845 970	LLGHT	**Os06g14070**	DUF581 domain containing protein, expressed	no
	6	7 848 384	7 854 067	LLGHT	**Os06g14080**	ATMAP70 protein, putative, expressed	no
	6	7 920 479	7 924 037	LLGHT	**Os06g14190**	NF-X1-type zinc finger protein, putative, expressed	**AT1G10170.1**
	6	7 925 697	7 927 406	LLGHT	**Os06g14200**	zinc finger, C3HC4 type domain containing protein, expressed	**AT4G24204.3**
	6	7 933 984	7 935 241	LLGHT	**Os06g14220**	STF-1, putative, expressed	no
	6	7 940 906	7 941 692	LLGHT	**Os06g14240**	hsp20/alpha crystallin family protein, putative, expressed	**AT5G37670.1**
	6	7 953 935	7 956 231	LLGHT	**Os06g14260**	lectin-like receptor kinase, putative, expressed	no
	6	7 963 879	7 967 790	LLGHT	**Os06g14280**	cysteine-rich repeat secretory protein precursor, putative, expressed	no
	6	7 982 084	7 983 859	LLGHT	**Os06g14310**	potassium channel AKT1, putative, expressed	no
	6	7 983 955	7 990 958	LLGHT	**Os06g14324**	caleosin related protein, putative, expressed	no
	6	7 996 891	8 004 842	LLGHT	**Os06g14350**	caleosin related protein, putative, expressed	no
	6	8 011 835	8 014 837	LLGHT	**Os06g14370**	caleosin related protein, putative, expressed	**AT1G70670.1**
	6	8 031 719	8 035 243	LLGHT	**Os06g14390**	1-aminocyclopropane-1-carboxylate oxidase homolog 4, putative, expressed	no
	6	8 038 363	8 041 831	LLGHT	**Os06g14400**	1-aminocyclopropane-1-carboxylate oxidase homolog 4, putative, expressed	no
	6	8 043 564	8 049 979	LLGHT	**Os06g14406**	SYD, putative, expressed	no
	6	8 068 591	8 070 044	LLGHT	**Os06g14420**	hydrolase, NUDIX family, domain containing protein, expressed	no
	6	8 097 281	8 104 472	LLGHT	**Os06g14440**	DRD1, putative, expressed	no
**q87**	**6**	**9 185 455**		**R_S**			
	6	13 996 414	13 997 319	DWB60	**Os06g23950**	OsMADS59 - MADS-box family gene with MIKCc type-box	no
	6	14 006 453	14 009 298	DWB60	Os06g23980	transcription factor, putative, expressed	no
**q76**	**6**	**14 008 461**	**16 515 631**	**DWB60**			
	6	14 078 258	14 082 303	DWB60	Os06g24070	myb-like DNA-binding domain containing protein, expressed	**AT2G20570.2**
**q41**	**6**	**14 126 219**		**NCR, DW0020, RDW**			
	6	15 840 814	15 842 660	DWB60	**Os06g27910**	oleosin, putative, expressed	no
	6	15 848 243	15 848 563	DWB60	**Os06g27920**	zinc ion binding protein, putative, expressed	no
	6	**15 863 214**	**15 872 704**	DWB60	**Os06g27970**	FKBP12-interacting protein of 37 kDa, putative, expressed	**AT3G54170.1**
	6	15 877 891	15 879 564	DWB60	**Os06g27980**	methyltransferase domain containing protein, expressed	**AT3G05390.1**
	6	16 774 133	16 785 410	DWB60	**Os06g29350**	myosin, putative, expressed	**AT1G08730.1**
**q77**	**6**	**16 798 522**		**DWB60**			
	6	16 896 515	16 897 661	DRW	**Os06g29470**	peroxidase precursor, putative, expressed	no
**q79**	**6**	**16 912 708**		**DRW**			
	6	17 003 628	17 010 231	DW2040	**Os06g29650**	CDP-diacylglycerol--inositol 3-phosphatidyltransferase 1, putative, expressed	no
**q68**	**6**	**17 005 559**	**17 036 530**	**DW2040**			
	6	17 035 080	17 036 915	DW2040	**Os06g29690**	dynein light chain type 1 domain containing protein, expressed	no
	6	17 044 170	17 046 351	DW2040	**Os06g29700**	OsFBD11 - F-box and FBD domain containing protein, expressed	no
	6	17 054 545	17 066 625	DW2040	**Os06g29710**	OsFBD12 - F-box and FBD domain containing protein, expressed	no
	6	19 866 070	19 870 006	NCR,MRL	**Os06g34120**	GDSL-like lipase/acylhydrolase, putative, expressed	no
**q35**	**6**	**19 870 050**	**20 145 257**	**NCR, MRL**			
	6	20 010 528	20 012 036	NCR	**Os06g34390**	zinc finger, C3HC4 type domain containing protein, expressed	no
	6	20 019 172	20 021 317	NCR	**Os06g34400**	zinc finger, C3HC4 type domain containing protein, expressed	no
	6	20 025 972	20 031 155	NCR	**Os06g34420**	DEAD/DEAH box helicase domain protein, putative, expressed	no
	6	20 033 279	20 034 343	NCR	**Os06g34430**	zinc finger protein, putative, expressed	no
	6	20 051 265	20 052 498	NCR	**Os06g34450**	zinc finger, C3HC4 type domain containing protein, expressed	no
	6	20 064 666	20 065 169	NCR	**Os06g34470**	zinc finger, C3HC4 type domain containing protein, expressed	no
	6	20 085 771	20 086 592	NCR	**Os06g34520**	CAF1 family ribonuclease containing protein, putative, expressed	no
	6	20 087 862	20 089 203	NCR	**Os06g34530**	zinc finger, C3HC4 type domain containing protein, expressed	no
	6	20 098 734	20 100 496	NCR	**Os06g34560**	zinc finger, C3HC4 type domain containing protein, expressed	no
	6	20 102 688	20 104 439	NCR	**Os06g34564**	mucin, putative	no
	6	20 131 644	20 132 168	NCR	**Os06g34620**	Zinc finger, C3HC4 type domain containing protein, expressed	no
	6	20 138 220	20 138 690	NCR	**Os06g34640**	zinc finger, C3HC4 type domain containing protein, expressed	no

QTL	Chr.	Site 1	Site 2	Traits	Gene code	Annotation	Arabidopsis
	6	20 143 793	20 144 666	NCR	**Os06g34650**	zinc finger, C3HC4 type domain containing protein, expressed	no
	6	20 145 118	20 148 099	NCR	**Os06g34660**	X8 domain containing protein, expressed	**AT3G58100.1**
	6	20 158 641	20 165 624	NCR	**Os06g34690**	T-complex protein, putative, expressed	**AT5G26360.1**
	6	21 692 500	21 692 819	DRP	**Os06g36820**	cysteine synthase, putative, expressed	no
	6	21 695 602	21 699 561	DRP	**Os06g36830**	cysteine synthase, mitochondrial precursor, putative, expressed	no
	6	21 700 478	21 703 430	DRP	**Os06g36840**	cysteine synthase, putative, expressed	no
	6	21 708 951	21 714 106	DRP	**Os06g36850**	cysteine synthase, putative, expressed	no
**q82**	**6**	**21 716 314**	**21 716 317**	**DRP**			
	6	21 730 402	21 733 637	DRP	**Os06g36880**	cysteine synthase, putative, expressed	no
	6	21 735 125	21 739 838	DRP	**Os06g36890**	glycosyl transferase, group 1 domain containing protein, expressed	no
	6	22 821 117	22 823 478	DEPTH	**Os06g38510**	pectate lyase precursor, putative, expressed	no
	6	22 824 676	22 826 319	DEPTH	**Os06g38520**	pectate lyase family protein, expressed	no
**q22**	**6**	**22 826 683**	**22 829 858**	**DEPTH**			
**q88**	**6**	**24 668 182**		**R_S**			
	6	30 161 499	30 162 653	DEPTH	**Os06g49830**	SHI, putative, expressed	**AT3G51060.1**
	6	30 173 485	30 178 066	DEPTH	**Os06g49840**	OsMADS16 - MADS-box family gene with MIKCc type-box, expressed	AT3G54340.1
**q23**	**6**	**30 176 431**		**DEPTH**			
	6	30 183 274	30 186 939	DEPTH	**Os06g49860**	methyltransferase, putative, expressed	**AT3G51070.1**
	6	30 187 080	30 189 238	DEPTH	**Os06g49870**	tRNA-dihydrouridine synthase, putative, expressed	**AT5G67220.1**
	6	30 196 097	30 197 605	DEPTH	**Os06g49880**	B-box zinc finger family protein, putative, expressed	no
	6	30 967 319	30 973 486	DEPTH	**Os06g51190**	lysine ketoglutarate reductase trans-splicing related 1, putative, expressed	no
	6	30 976 066	30 981 070	DEPTH	**Os06g51200**	lysine ketoglutarate reductase trans-splicing related 1, putative, expressed	no
	6	30 986 739	30 988 361	DEPTH	**Os06g51210**	zinc finger family protein, putative, expressed	no
	6	30 993 254	30 995 283	DEPTH	**Os06g51220**	HMG1/2, putative, expressed	no
**q24**	**6**	**30 995 770**		**DEPTH**			
	6	31 013 731	31 021 754	DEPTH	**Os06g51250**	EF hand family protein, putative, expressed	no
	7	446 619	451 162	NCR	**Os07g01760**	aminotransferase, classes I and II, domain containing protein, expressed	**AT1G70580.1**
	7	457 738	460 218	NCR	**Os07g01780**	GTPase activating protein, putative, expressed	no
	7	464 358	465 341	NCR	**Os07g01800**	cysteine proteinase EP-B 1 precursor, putative, expressed	no
	7	467 286	468 754	NCR	**Os07g01810**	potassium channel protein, putative, expressed	no
	7	471 174	477 655	NCR	**Os07g01820**	OsMADS15 - MADS-box family gene with MIKCc type-box, expressed	no
**q42**	**7**	**474 875**		**NCR**			
	7	15 406 442	15 409 801	THK	**Os07g26690**	aquaporin protein, putative, expressed	no
	**7**	**15 421 670**	15 425 213	**THK**	**Os07g26700**	UPF0041 domain containing protein, putative, expressed	**AT4G22310.1**
**q61**	**7**	**15 424 576**		**THK**			
	7	15 440 889	15 442 180	THK	**Os07g26720**	OsRR7 type-A response regulator, expressed	no
	7	15 448 959	15 451 104	THK	**Os07g26740**	60S ribosomal protein L44, putative, expressed	no
	7	17 186 673	17 187 640	NR_T	**Os07g29310**	OsSAUR30 - Auxin-responsive SAUR gene family member, expressed	no
	7	17 195 209	17 196 988	NR_T	**Os07g29320**	rRNA-processing protein EBP2, putative, expressed	**AT3G22660.1**
	7	**17 198 415**	**17 209 485**	NR_T	**Os07g29330**	serine/threonine-protein kinase CTR1, putative, expressed	no
**q52**	**7**	**17 207 987**		**NR_T**			
	7	17 230 234	17 245 358	NR_T	**Os07g29360**	tetratricopeptide repeat domain containing protein, expressed	no
	7	18 479 043	18 481 052	SDW, PDW	**Os07g31210**	OsWAK70 - OsWAK receptor-like cytoplasmic kinase OsWAK-RLCK, expressed	no
	7	18 497 999	18 502 813	SDW, PDW	**Os07g31250**	OsWAK69 - OsWAK receptor-like cytoplasmic kinase OsWAK-RLCK, expressed	no
**q15**	**7**	**18 507 925**		**SDW, PDW**			
	7	18 509 977	18 515 210	SDW, PDW	**Os07g31270**	cupin 2, conserved barrel domain protein, putative, expressed	**AT4G17050.1**
	7	18 519 358	18 523 619	SDW, PDW	**Os07g31280**	casein kinase II subunit beta-4, putative, expressed	no
	7	18 528 508	18 531 797	SDW, PDW	**Os07g31290**	tyrosine protein kinase domain containing protein, putative, expressed	no
	7	18 532 539	18 535 080	SDW, PDW	**Os07g31300**	ATP synthase delta chain, mitochondrial precursor, putative, expressed	no
	7	20 803 306	20 805 476	TIL	**Os07g34710**	peroxidase precursor, putative, expressed	no
	7	20 806 654	20 807 546	TIL	**Os07g34720**	harpin-induced protein 1 domain containing protein, expressed	**AT4G05220.1**
	7	20 808 033	20 812 544	TIL	**Os07g34730**	acyltransferase, putative, expressed	no
	7	20 812 882	20 817 921	TIL	**Os07g34740**	NUC153 domain containing protein, expressed	**AT3G01160.1**
**q12**	**7**	**20 825 918**		**TIL**			
	7	20 842 619	20 845 992	TIL	**Os07g34780**	ankyrin repeat domain-containing protein, putative, expressed	no
	7	22 169 511	22 171 932	NR_T	**Os07g37010**	cyclin, putative, expressed	**AT4G03270.1**
**q53**	**7**	**22 174 085**	**22 175 036**	**NR_T**			
	7	22 184 376	22 187 030	NR_T	**Os07g37030**	cytochrome b6-f complex iron-sulfur subunit, chloroplast precursor, putative,	**AT4G03280.1**
	7	22 187 290	22 189 082	NR_T	**Os07g37040**	PB1 domain containing protein, expressed	**AT4G05150.1**
	7	29 458 303	29 459 301	DEPTH	**Os07g49200**	membrane associated DUF588 domain containing protein, putative, expressed	no
	7	**29 465 173**	**29 475 499**	DEPTH	**Os07g49210**	helicase conserved C-terminal domain containing protein, expressed	**AT3G42670.1**
**q25**	**7**	**29 468 499**		**DEPTH**			
	7	29 478 758	29 483 659	DEPTH	**Os07g49230**	ubiquitin-activating enzyme, putative, expressed	no
	7	29 484 521	29 487 438	DEPTH	**Os07g49240**	MRH1, putative, expressed	no
	7	29 490 011	29 492 173	DEPTH	**Os07g49250**	thiamine pyrophosphate enzyme, C-terminal TPP binding domain containing	no
	8	4 385 768	4 389 031	LLGHT	**Os08g07830**	DUF292 domain containing protein, expressed	**AT1G34220.1**
	8	4 390 729	4 400 757	LLGHT	**Os08g07840**	PolI-like DNA polymerase, putative, expressed	**AT1G50840.1**
	8	4 407 120	4 416 924	LLGHT	**Os08g07850**	PolI-like DNA polymerase, putative, expressed	no
**q3**	**8**	**4 413 495**		**LLGHT**			
	8	4 430 196	4 434 313	LLGHT	**Os08g07880**	phosphopantothenate--cysteine ligase, putative, expressed	no
	8	4 523 651	4 532 056	THK	**Os08g08000**	DNA binding protein, putative, expressed	**AT3G24870.1**
**q62**	**8**	**4 544 057**		**THK**			
	8	4 549 380	4 557 530	THK	**Os08g08030**	double-strand break repair protein MRE11, putative, expressed	no
	8	4 562 428	4 565 405	THK	**Os08g08040**	LSM domain containing protein, expressed	**AT2G03870.2**
	8	15 476 556	15 483 069	DEPTH	**Os08g25430**	cysteine-rich receptor-like protein kinase 35 precursor, putative, expressed	no
	8	15 494 238	15 501 850	DEPTH	**Os08g25460**	transcription initiation factor, putative, expressed	**AT1G03280.1**
**q26**	**8**	**15 504 028**	**15 546 812**	**DEPTH**			
	8	15 510 426	15 512 115	DEPTH	**Os08g25490**	berberine and berberine like domain containing protein, expressed	no
	8	27 380 765	27 390 339	SDW	**Os08g43334**	HSF-type DNA-binding domain containing protein, expressed	no
	8	27 393 219	27 396 113	SDW	**Os08g43350**	NC domain-containing protein, putative, expressed	no
	8	27 397 948	27 402 221	SDW	**Os08g43360**	RNA recognition motif containing protein, putative, expressed	**AT1G21320.1**
	8	27 403 439	27 406 438	SDW	**Os08g43370**	6-phosphogluconolactonase, putative, expressed	no
	8	27 408 976	27 413 886	SDW	**Os08g43380**	TBC domain containing protein, expresse	no

QTL	Chr.	Site 1	Site 2	Traits	Gene code	Annotation	Arabidopsis
**q16**	**8**	**27 413 965**		**SDW**			
	8	27 420 501	27 422 836	SDW	**Os08g43390**	cytochrome P450, putative, expressed	**AT3G61880.2**
	8	27 435 126	27 442 742	SDW	**Os08g43400**	kinesin motor domain containing protein, expressed	no
	10	11 581 250	11 585 288	DW4060, D	**Os10g22380**	glutamyl-tRNA synthetase, cytoplasmic, putative, expressed	**AT5G26710.1**
**q70**	**10**	**11 589 746**		**DW4060, DRW**			
	10	11 596 532	11 599 845	DW4060, D	**Os10g22410**	yippee zinc-binding protein, putative, expressed	no
	10	11 605 114	11 609 036	DW4060, D	**Os10g22430**	gibberellin response modulator protein, putative, expressed	**AT5G48150.1**
	10	11 691 142	11 695 039	DEPTH	**Os10g22560**	peptide transporter PTR2, putative, expressed	no
	10	**11 698 992**	**11 713 558**	DEPTH	**Os10g22570**	cellulase, putative, expressed	no
**q27**	**10**	**11 712 638**		**DEPTH**			
	10	11 723 419	11 726 426	DEPTH	**Os10g22590**	POEI24 - Pollen Ole e I allergen and extensin family protein precursor, expressed	**AT4G08685.1**
	10	11 731 592	11 732 917	DEPTH	**Os10g22600**	ethylene-responsive transcription factor, putative, expressed	no
	10	15 290 345	15 292 947	DEPTH	**Os10g29430**	RNA-directed DNA polymerase, putative, expressed	no
	10	15 294 053	15 295 204	DEPTH	**Os10g29440**	speckle-type POZ protein, putative, expressed	no
	10	**15 303 901**	**15 309 302**	DEPTH	**Os10g29470**	dehydrogenase, putative, expressed	**AT4G37970.1**
**q28**	**10**	**15 307 568**		**DEPTH**			
	10	15 324 463	15 331 200	DEPTH	**Os10g29495**	speckle-type POZ protein, putative, expressed	no
**q43**	**11**	**5 272 788**		**NCR**			
	11	5 282 058	5 284 700	NCR	**Os11g09864**	wali7, putative, expressed	no
	11	7 877 895	7 879 548	NCR	**Os11g14140**	OsFBK25 - F-box domain and kelch repeat containing protein, expressed	**AT2G44130.1**
	11	7 909 213	7 917 123	NCR	**Os11g14170**	OsDegp12 - Putative Deg protease homologue, expressed	**AT5G27660.1**
**q44**	**11**	**7 927 995**		**NCR**			
	11	7 938 345	7 939 755	NCR	**Os11g14190**	ARGOS, putative, expressed	no
	11	8 948 771	8 951 203	NCR, DW00	**Os11g16260**	laccase precursor protein, putative, expressed	no
	11	8 965 406	8 970 029	NCR, DW00	**Os11g16280**	F-box domain containing protein, expressed	**AT3G07550.1**
	11	8 970 074	8 977 435	NCR, DW00	**Os11g16290**	NIN, putative, expressed	**AT2G43500.1**
**q45**	**11**	**8 972 097**	**9 004 279**	**NCR, DW0020, DW2040, RDW, PDW**			
**q46**	**11**	**16 559 637**		**NCR**			
	11	16 917 660	16 919 756	TIL	**Os11g29190**	40S ribosomal protein S5, putative, expressed	**AT2G37270.1**
	11	16 926 681	16 937 157	TIL	Os11g29200	protein transport protein, putative, expressed	**AT3G44340.1**
**q13**	**11**	**16 929 527**		**TIL**			
	11	16 937 492	16 939 353	TIL	**Os11g29210**	resistance-gene-interacting protein, putative, expressed	no
	11	16 943 497	16 946 706	TIL	**Os11g29230**	pentatricopeptide repeat protein PPR986-12, putative, expressed	no
	11	17 108 744	17 115 599	THK	**Os11g29490**	plasma membrane ATPase, putative, expressed	no
**q63**	**11**	**17 111 220**	**17 318 688**	**THK**			
	11	17 117 873	17 121 629	THK	**Os11g29510**	cysteine-rich receptor-like protein kinase 35 precursor, putative, expressed	no
	11	17 126 630	17 132 216	THK	**Os11g29520**	NBS-LRR d	no
	11	17 169 023	17 169 357	THK	**Os11g29600**	RING zinc finger ankyrin protein, putative, expressed	no
	11	17 208 164	17 210 164	THK	**Os11g29690**	oxidoreductase, 2OG-Fe oxygenase family protein, putative, expressed	AT1G11780.1
	11	17 229 938	17 231 917	THK	**Os11g29710**	OsFBO4 - F-box and other domain containing protein, expressed	no
	11	17 234 285	17 238 178	THK	**Os11g29720**	cytochrome P450, putative, expressed	no
	11	17 259 927	17 264 943	THK	**Os11g29750**	plasma membrane ATPase, putative, expressed	no
	11	17 284 028	17 285 405	THK	**Os11g29780**	plant-specific domain TIGR01627 family protein, expressed	**AT1G67330.1**
	11	17 291 100	17 294 288	THK	**Os11g29790**	receptor kinase, putative, expressed	no
	11	17 329 608	17 332 992	THK	**Os11g29850**	ABC transporter, ATP-binding protein, putative, expressed	**AT5G14100.1**
**q29**	**11**	**17 843 772**	**17 858 468**	**DEPTH**			
	11	18 092 965	18 095 638	DEPTH	**Os11g31090**	transferase family protein, putative, expressed	**AT5G41040.1**
	**11**	18 096 831	18 102 879	DEPTH	**Os11g31100**	gibberellin response modulator protein, putative, expressed	no
**q30**	**11**	**18 101 744**		**DEPTH**			
	11	22 550 213	22 554 890	DEPTH	**Os11g38010**	targeting protein for Xklp2, putative, expressed	no
	11	22 555 514	22 561 050	DEPTH	**Os11g38020**	GTPase of unknown function domain containing protein, putative, expressed	**AT5G57960.1**
	11	22 561 543	22 565 402	DEPTH	**Os11g38030**	CDK5RAP1, putative, expressed	**AT4G36390.1**
	11	22 569 981	22 576 763	DEPTH	**Os11g38050**	phosphoesterase family protein, putative, expressed	no
**q31**	**11**	**22 579 249**		**DEPTH**			
	11	22 600 678	22 602 126	DEPTH	**Os11g38100**	OsFBDUF55 - F-box and DUF domain containing protein, expressed	no
	12	1 157 980	1 165 291	DW4060	**Os12g03080**	rp3 protein, putative, expressed	no
	12	1 169 472	1 170 473	DW4060	**Os12g03090**	ribosomal protein, putative, expressed	**AT2G09990.1**
	12	1 172 671	1 175 788	DW4060	**Os12g03100**	exostosin family domain containing protein, expressed	no
	12	1 180 358	1 181 099	DW4060	**Os12g03110**	ZF-HD protein dimer	no
**q71**	**12**	**1 185 335**		**DW4060**			
	12	1 189 043	1 191 517	DW4060	**Os12g03130**	CDC45A - Putative DNA replication initiation protein, expressed	no
	12	1 200 471	1 202 202	DW4060	**Os12g03150**	myb-like DNA-binding domain containing protein, putative, expressed	**AT1G08810.1**
	12	1 305 139	1 307 108	NCR	**Os12g03360**	EMB1379, putative, expressed	no
	12	1 309 401	1 310 704	NCR	**Os12g03370**	harpin-induced protein 1 domain containing protein, expressed	no
	12	1 315 753	1 317 248	NCR	**Os12g03390**	OsFBX435 - F-box domain containing protein, expressed	no
**q47**	**12**	**1 323 261**		**NCR**			
	12	1 332 836	1 333 568	NCR	**Os12g03420**	VQ domain containing protein, putative, expressed	no
	12	1 337 191	1 339 507	NCR	**Os12g03430**	Mpv17 / PMP22 family domain containing protein, expressed	no
	12	1 342 002	1 344 257	NCR	**Os12g03440**	OsFBX436 - F-box domain containing protein, expressed	no
	12	7 664 708	7 669 593	DEPTH	**Os12g13640**	glycosyltransferase, putative, expressed	no
**q32**	**12**	**7 681 309**		**DEPTH**			
	12	25 116 029	25 119 760	TIL	**Os12g40590**	helix-loop-helix DNA-binding domain containing protein, expressed	no
**q14**	**12**	**25 142 679**		**TIL**			
	12	25 142 777	25 148 196	TIL	**Os12g40630**	helix-loop-helix DNA-binding domain containing protein, expressed	no
	12	27 316 455	27 317 290	NR_T	**Os12g44050**	purple acid phosphatase precursor, putative, expressed	no
	12	27 324 604	27 327 502	NR_T	**Os12g44060**	nodulin, putative, expressed	no
	12	27 329 996	27 332 079	NR_T	**Os12g44070**	nodulin, putative, expressed	no
	12	27 336 089	27 337 780	NR_T	**Os12g44080**	CHCH domain containing protein, expressed	no
**q54**	**12**	**27 338 111**		**NR_T**			
	12	27 340 225	27 344 135	NR_T	**Os12g44090**	leucine-rich repeat family protein, putative, expressed	**AT5G62710.1**
	12	27 346 122	27 350 696	NR_T	**Os12g44100**	peptide transporter PTR2, putative, expressed	no
	12	27 359 424	27 361 454	NR_T	**Os12g44110**	ligA, putative, expressed	no
